# The role of primed and non-primed MSC-derived conditioned media in neuroregeneration

**DOI:** 10.3389/fnmol.2023.1241432

**Published:** 2023-11-02

**Authors:** Nikola Hudakova, Dagmar Mudronova, Dana Marcincakova, Lucia Slovinska, Petra Majerova, Marcela Maloveska, Patricia Petrouskova, Filip Humenik, Dasa Cizkova

**Affiliations:** ^1^Centre of Experimental and Clinical Regenerative Medicine, University of Veterinary Medicine and Pharmacy in Kosice, Košice, Slovakia; ^2^Department of Microbiology and Immunology, University of Veterinary Medicine and Pharmacy in Kosice, Košice, Slovakia; ^3^Department of Pharmacology and Toxicology, University of Veterinary Medicine and Pharmacy in Kosice, Košice, Slovakia; ^4^Associated Tissue Bank, Faculty of Medicine, Pavol Jozef Safarik University and Luis Pasteur University Hospital, Košice, Slovakia; ^5^Institute of Neuroimmunology, Slovak Academy of Sciences, Bratislava, Slovakia

**Keywords:** MSC, conditioned medium, IFN-γ, neurotrophic effect, priming

## Abstract

**Introduction:**

With growing significance in nervous system repair, mesenchymal stem cell-derived conditioned media (MSCCM) have been used in cell-free therapies in regenerative medicine. However, the immunomodulatory and neuroregenerative effects of MSCCM and the influence of priming on these effects are still poorly understood.

**Methods:**

In this study, by various methods focused on cell viability, proliferation, neuron-like differentiation, neurite outgrowth, cell migration and regrowth, we demonstrated that MSCCM derived from adipose tissue (AT-MSCCM) and amniotic membrane (AM-MSCCM) had different effects on SH-SY5Y cells.

**Results and discussion:**

AT-MSCCM was found to have a higher proliferative capacity and the ability to impact neurite outgrowth during differentiation, while AM-MSCCM showed more pronounced immunomodulatory activity, migration, and re-growth of SH-SY5Y cells in the scratch model. Furthermore, priming of MSC with pro-inflammatory cytokine (IFN-γ) resulted in different proteomic profiles of conditioned media from both sources, which had the highest effect on SH-SY5Y proliferation and neurite outgrowth in terms of the length of neurites (pAT-MSCCM) compared to the control group (DMEM). Altogether, our results highlight the potential of primed and non-primed MSCCM as a therapeutic tool for neurodegenerative diseases, although some differences must be considered.

## 1. Introduction

Mesenchymal stromal perivascular cells (MSC) are multipotent, plastic-adherent cells with self-renewal capacity that may be collected from a variety of sources. The International Society for Cellular Therapy (ISCT) has established specific classification criteria for these cells. They must be plastic adherent, be able to differentiate into bone, cartilage, and adipose tissue, and express the surface markers CD105, CD73, and CD90. They must not express CD45, CD34, CD14, CD11b, CD79 alpha, or CD19 (Lindroos et al., 2011; Gugliandolo and Mazzon, 2022).

MSC have been isolated from various tissues. In clinical trials, bone marrow (BM) is the most often used source of MSC, followed by the umbilical cord (UC) and adipose tissue (AT) (Jovic et al., 2022). With well-characterized properties, bone marrow-derived mesenchymal stem cells (BM-MSC) have long been regarded the “gold standard” for cell treatment (Ahmadbeigi et al., 2011). However, although the fact that BM harvesting can be a painful and invasive procedure for the donor and the cell yield, as well as cell lifespan, proliferative capacity, and differentiation potential, decreases with increasing donor age, it is still an important source of stem cells for transplantation in certain medical conditions (Zaim et al., 2012).

On the contrary, AT is more abundant and readily available throughout the body than BM, making it a more convenient and accessible source of MSC, particularly during therapeutic liposuction procedures or abdominal surgeries. It is estimated that approximately 98–100% of cells obtained from AT remain viable after isolation (Berebichez-Fridman and Montero-Olvera, 2018). In addition to their stability in long-term cell cultures, adipose tissue-derived MSC (AT-MSC) have a high potential for multilineage differentiation and can expand successfully *in vitro*. Therefore, compared to BM, AT is a more useful autologous source of MSC for tissue engineering (Choudhery et al., 2014; Coelho et al., 2020).

Amniotic membrane-derived mesenchymal stem cells (AM-MSC) with similar immune phenotype and multilineage differentiation potential as MSC derived from previously mentioned sources have higher expansivity in comparison with BM-MSC and AT-MSC (Berebichez-Fridman and Montero-Olvera, 2018). The use of perinatal tissues (AM, placental membrane, UC) in research and cell therapy is not ethically problematic because they are regarded as medical waste (Udalamaththa et al., 2020). *In vitro*, AM-MSC displayed immunomodulatory ability, which is comparable to BM-MSC properties (Yun Cheng, 2014).

Accumulating evidence suggests that the pleiotropic effects of MSC are not due to their differentiation abilities but rather mediated by the releases of soluble bioactive molecules (also called secretome) (Yari et al., 2022). Significant ones include promoting neuroprotection, neurogenesis, and angiogenesis, as well as preventing neuroinflammation (Lo Furno et al., 2018). Neurotrophic factors (NTFs), such as glial cell-derived neurotrophic factor (GDNF), nerve growth factor (NGF), and brain-derived neurotrophic factor (BDNF), as well as the production of anti-inflammatory molecules such as transforming growth factor (TGF), interleukin-10 (IL-10), and tumor necrosis factor alpha-stimulated gene-6 (TSG-6), are primarily responsible for these effects (Yari et al., 2022).

During the last decades, researchers have sought different strategies to improve the therapeutic effects of MSC and MSCCM (Park et al., 2023). Preconditioning of MSC with the inflammatory cytokines (e.g., interferon gamma, IFN-γ) enhances their immunosuppressive activities as well as their expression of HLA and proinflammatory genes (Noone et al., 2013; Park et al., 2023). Accordingly, MSCCM both primed with IFN-γ (pMSCCM) and non-primed have become a promising novel cell-free approach. Clinical trials have demonstrated the safety and efficacy of MSC-free therapies for various neurological disorders, including spinal cord and brain injuries, stroke, and multiple sclerosis (Kvistad et al., 2022). However, further research is needed to fully understand the mechanisms underlying the neuroprotective effect of MSCCM and to optimize their therapeutic potential.

Therefore, in our study, we evaluated the neurotrophic and immunomodulatory potential of MSCCM derived from adult (adipose tissue, AT-MSCCM) and perinatal (amniotic membrane, AM-MSCCM) tissues. We also investigated whether we can enhance the production of bioactive molecules by priming MSC with an inflammatory cytokine IFN-γ. Using liquid chromatography-mass spectrometry (LC/MS–MS), we analyzed the secretory profile of all conditioned media (AT-MSCCM, AM-MSCCM) primed with IFN-γ and non-primed. By evaluating the expression of specific pro and anti-inflammatory markers (Luminex assay), we gained insights into how MSCCM (primed or non-primed) may affect the immune response in different experimental conditions. Finally, we assessed the efficacy and functionality of these factors by testing the impact of conditioned media on metabolic activity, proliferation, differentiation, an outgrowth of neurites-number, length, and re-growth using neuroblastoma cell line SH-SY5Y.

## 2. Materials and methods

### 2.1. MSC isolation

#### 2.1.1. AM-MSC isolation

Amniotic membranes were taken after cesarean section from healthy newborn dogs (*n* = 8), and thoroughly washed in a solution containing PBS (phosphate buffered saline, Sigma, United States), penicillin (300 U/mL) and streptomycin (300 μg/mL) (ATB/ATM, Sigma, United States). Individual tissues were mechanically divided into smaller pieces and incubated with a minimum of 0.05% collagenase solution (Collagenase type IV, Gibco, United States) for 15 min at 37° C. After incubation, amniotic tissue was filtered through a 100 μm cell strainer, centrifuged (1,200 rpm /7 min). The cell pellet was resuspended in a medium consisting of Dulbecco’s modified Eagle’s medium High Glucose (DMEM HG, Sigma, United States), 10% FBS (Sigma, United States), penicillin (100 U/mL), streptomycin (100 μg/mL), amphotericin B (2.5 μg/mL) and gentamicin (5 μg/mL) (Lonza, Switzerland), and subsequently incubated at 37° C and 5% CO_2_ in culture flasks T25 cm^2^/T75 cm^2^ in the concentration of 0.75×10^6^ for T25 cm^2^ and 2×10^6^ for T75 cm^2^ flask. Non-adherent cells are removed after 24 h and the medium was changed to fresh every 2–3 days as needed.

#### 2.1.2. AT-MSC isolation

The dorsal scapular region of purebred dogs (*n* = 5) was used to collect subcutaneous fat. Adipose tissue was washed with 2% ATB/ATM in PBS. Collagenase type I (Gibco, United States) was used to enzymatically separate the dissected tissue for 45 min at 37°C. To remove the remaining fragments of digested tissue after incubation, the digested tissue was run through a 100 μm cell strainer. The obtained fraction was centrifuged (1,200 rpm /7 min). The pellet with stromal vascular fraction (SVF) was resuspended in DMEM HG supplemented with 10% FBS and 2% antibiotics and then plated in the concentration of 0.75 × 10^6^ on 25 cm^2^ tissue culture flask. After two to three days, non-adherent cells were removed, and the culture media was replaced twice a week.

### 2.2. Passaging

Passaging was performed by a cell confluence of about 80% after seven days by the addition of 0.25% trypsin (Trypsin–EDTA) for 3–5 min at 37°C, its subsequent inactivation with the same volume of 10% FBS, followed by centrifugation of the cells in the obtained suspension for 7 min at 1200 rpm and by transferring the dissociated pellet to new adequate culture flasks.

### 2.3. Cryopreservation

The stem cells were stored in a cryopreservation medium, consisting of 50% FBS, 40% DMEM HG and 10% DMSO (dimethyl sulfoxide, Sigma, United States). After initial gentle freezing of the cells in a portable freezing container (Nalgene-Mr. Frosty Freezing Container) for 24 in −80°C freezer, the cryovials were transferred to liquid nitrogen.

### 2.4. Multilineage profile of AM-MSC and AT-MSC

The multilineage potential (osteogenic, chondrogenic, and adipogenic phenotypes) of canine AM-MSC and AT-MSC (both P3) was determined by incubation with commercial StemPro Differentiation Kits containing all the reagents required for inducing canine AM-MSC and AT-MSC into osteogenic, chondrogenic and adipogenic lineages. Cells were induced with the specific differentiation medium for 21 days according to the recommended protocol for each lineage. The cells after fixation (4% paraformaldehyde) were stained with the following agents (all from Sigma-Aldrich, United States): osteogenic culture with Alizarin Red S, chondrogenic culture with Alcian Blue, and adipogenic culture with Oil Red O.

### 2.5. Flow cytometry

AM-MSC and AT-MSC both in P3 were analyzed with commercially available antibodies to investigate the presence of the CD29, CD44, CD90-positive, and CD34, CD45-negative cells. Fluorochrome-conjugated monoclonal antibodies anti-CD29 (phycoerythrin, PE), anti-CD34 (PE), anti-CD44 (allophycocyanin, APC), anti-CD45 (PE), anti-CD90 (APC) diluted in PBS (all from Sigma, United States) were incubated with cell suspensions for 60 min at room temperature prevented from light. The cells were incubated, then washed twice in PBS before being centrifuged for five minutes at 1200 rpm. Followingly, PBS was added and cytometric measurements were performed using a BD FACSCanto™ flow cytometer (Becton Dickinson Biosciences, United States), and analyzed by BD FACS DivaTM analysis software, after gating the cells, the doublets and aggregates were eliminated, data are displayed as histograms.

### 2.6. Preparation of MSCCM and priming with IFN-γ

To obtain MSCCM (AT-MSCCM, AM-MSCCM), MSC were cultured in DMEM HG supplemented with 2% antibiotic-antimycotic solution (Sigma, United States) of ATB/ATM without the addition of FBS. MSC in the different passages (P1 - P4) at concentration 1.2 × 10^6^ were placed on a T75 culture flask in a standard culture medium (DMEM HG, 10% FBS, 2% antibiotic-antimycotic solution). After 48–72 h with 80% cell confluence, the medium was removed, the cells were washed twice with PBS and 5 mL of DMEM HG with 2% ATB/ATM without FBS was added.

In primed MSCCM (pAT-MSCCM, pAM-MSCCM) we added IFN-γ at a final concentration of 100 ng / mL. Finally, the conditioned medium was collected after 24 h, centrifuged twice for 7 min at 1200 rpm to remove cell debris, filtered through 0.2 μm sterilizing grade filter membrane and then frozen at −80°C until the time of use.

### 2.7. Immunomodulatory and proteomic analyses of MSCCM

**Figure 1:**
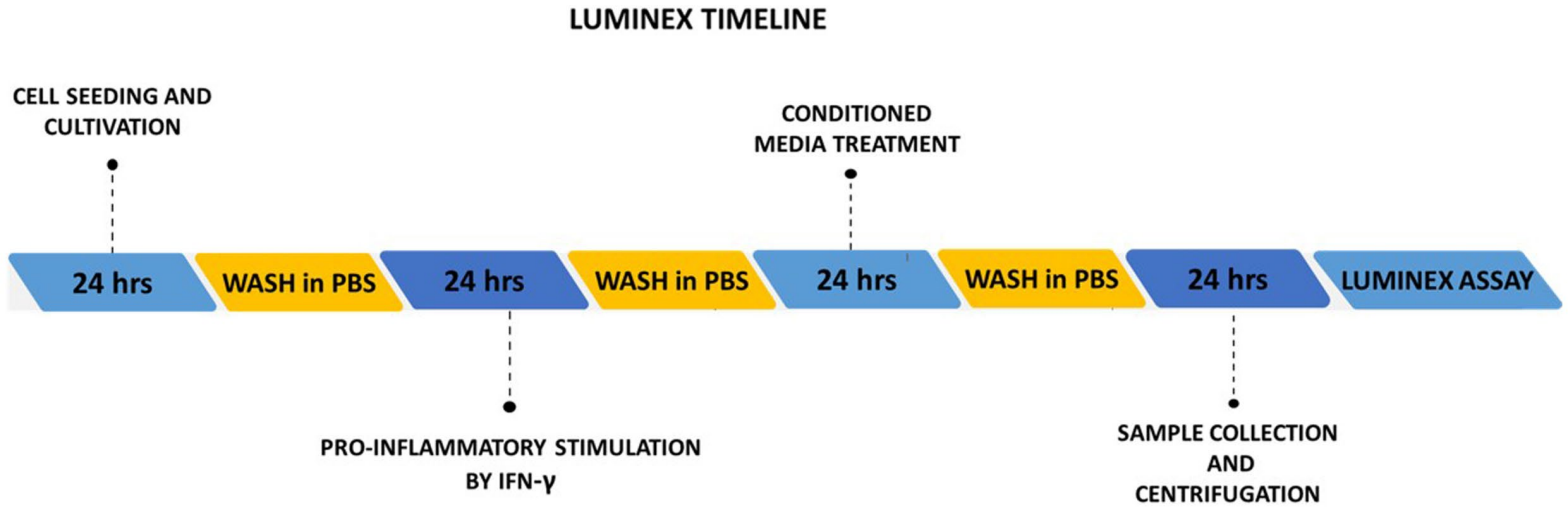
Schematic illustration of the experimental timelines of Luminex assay.

SH-SY5Y cells were seeded on 12-well plates at the density of cells per well (0.1×10^6^) and cultured in DMEM HG, 10% FBS, 2% ATB/ATM for 24 h, next day cells were washed in PBS solution and stimulated with pro-inflammatory cytokine IFN-γ (100 ng/mL, RND systems, GB) for 24 h. Afterward, the cells were washed in PBS and treated with conditioned media (AT-MSCCM, AM-MSCCM, pAT-MSCCM, pAM-MSCCM) for 24 h. SH-SH5Y treated with DMEM HG and 2% ATB/ATM for 24 h was considered as a control. Finally, cells were washed with PBS and cultured in DMEM HG for 24 h, after that media were collected, centrifuged at 1200 rpm for 10 min, and used for Luminex analysis (Figure 1). With this method we evaluated the levels of paracrine factors (IL-2, IL-6, IL-8, IL-10, IL-12, MCP-1) involved in inflammation by using magnetic bead technology from Luminex with the ProcartaPlex™ Canine Cytokine Chemokine Growth Factor (Thermo Fisher, United States), according to the manufacturer’s instructions. For each sample, the measurement was performed twice using MAGPIX Luminex to measure the analytes. Both Bio-Plex Manager 6.1 (Bio-Rad Laboratories, Hercules, CA, United States) and XPONENT software version 4.2 for MAGPIX (Luminex Corporation, Austin, TX, United States) were used for data analysis. After creating a standard curve, concentrations were extrapolated for each sample and expressed as pg./mL.

### 2.8. Proteomic analysis of MSCCM

From collected samples (AT-MSCCM, AM-MSCCM, pAT-MSCCM and pAM-MSCCM) 200 μL of media was precipitated by ice-cold 80% acetone overnight. Samples were centrifuged at 30000 g for 30 min. Pellets were dissolved in 8 M urea in Tris–HCl (ph = 8). The Bio-Rad protein assay was used to determine the total protein concentration (Bio-Rad Laboratories GmbH, Colbe, Germany). Hundred micrograms of proteins were reduced with 10 mM dithiothreitol (Sigma, United States) in 100 mM ammonium bicarbonate (Sigma-Aldrich, MO, United States) at 37°C for 60 min. Alkylation was carried out in the dark for 30 min with 15 μL of 500 mM iodoacetamide (Sigma, United States) in 100 mM ammonium bicarbonate. Proteins were digested overnight at 37°C with trypsin (Promega, United States) in a 1:100 ratio. 100 ng aliquots of pure complex peptide mixtures were separated using an Acquity M-Class UHPLC (Waters, Milford, United States). Samples were put into the 25 mm long, 180 mm diameter, and 5 m particle size nanoEase Symmetry C18 trap column and after 2 min of desalting, peptides were added to the nanoEase HSS T3 C18 analytical column (250 mm length, 75 m diameter, 1.8 m particle size) using an 8 L/min flow rate of 1% acetonitrile containing 0.1% formic acid. A 90-min gradient of 5–35% acetonitrile with 0.1% formic acid at a flow rate of 300 nL/min was used to achieve full separation. The samples were sprayed (3.1 kV capillary voltage) onto the Synapt G2-Si quadrupole time-of-flight mass spectrometer (Waters, Milford, United States). Progenesis QI 4.0 was used to process the data (Waters, United States). Using correlations of chroma-tographic elution profiles in low/high energy traces, precursors and fragment ions were coupled. After aligning peak retention periods on chromatograms, the peak intensities were converted to the median distribution of all ions and normalized.

### 2.9. XTT assay

SH-SY5Y cells (2 × 10^3^ cells/cm^2^) were seeded in the wells of a 96-well plate and cultured for 1, 2, and 4 days in DMEM HG medium supplemented with 3% FBS and 2% ATB/ATM for the control group and conditioned media (AM-MSCCM, pAM-MSCCM, AT-MSCCM, pAT-MSCCM supplemented with 3% FBS and 2% ATB/ATM) for experimental groups. Then, cellular metabolic activity was assessed by XTT using Cell Proliferation Kit II (XTT) according to the manufacturer’s instructions (Roche, Germany). DMEM HG with 2% ATB/ATM served as Blank. The absorbance (492 nm) was measured by APOLLO Absorbance Reader (Berthold Systems, United States).

### 2.10. xCELLigence real-time cell analysis (RTCA)

To investigate the effect of MSC-derived conditioned media with and without priming by IFN-γ on the proliferation of human SH-SY5Y cells RTCA was used. The main concept centers on the use of the electronic impedance technology detection method, measuring changes in a sample’s electrical properties, such as changes in conductivity. The presence of the cell membrane that comes into contact with the electrode and the strength of the adhesion alter the electrical impedance of the golden electrodes on the bottom of the E-plate surface (Miłek et al., 2019; Zhang et al., 2019). SH-SY5Y cells (2 × 10^3^ cells in 150 μL medium/well) were seeded in 16 well plates (E-plate 16, Roche, Mannheim, Germany) and cultured in MSCCM (AM-MSCCM, pAM-MSCCM, AT-MSCCM, pAT-MSCCM) supplemented with 2% FBS and 2% ATB/ATM following the xCELLigence Real-Time Cell Analyzer (RTCA, ACEA bio-sciences, United States) DP instrument manual as provided by the manufacturer (Real Time Cell Analyzer–RTCA, ACEA Biosciences, USA). The medium was replaced with new after 48 h, and a total of 120 h were given for the experiment to run. SH-SY5Y cultured in DMEM HG, 2% FBS, and 2% ATB/ATM were used as negative control and DMEM HG medium was considered as a blank.

### 2.11. SH-SY5Y neuron-like differentiation

Neuron-like differentiation was induced by following differentiating protocol. SH-SY5Y cells (1 × 10^4^ cells/cm^2^) in DMEM HG with 5% FBS were grown on coverslips (15 mm in diameter) previously coated with PureCol Bovine Collagen Solution Type I (Advanced BioMatrix) for 60 min and inserted in a 24-well plate. After three days, the media was changed to DMEM HG with 2% FBS and 10 mM all-trans-retinoic acid (RA; Sigma-Aldrich, United States, R2625), and the culture continued for an additional 5 days. The cells were then grown for another 5 days using Neurobasal medium (Gibco, United States) supplemented with 50 ng/mL BDNF (Thermo Fisher, United States). SH-SY5Y cells can differentiate into neuron-like cells, undergo morphological changes, express neuron-specific proteins, and acquire functional properties of mature neurons, such as the ability to form synapses and generate action potentials.

To assess the effect of MSCCM on neuron-like differentiation of SH-SY5Y, cells cultured on coverslips were cultured for 5 days in conditioned media (AT-MSCCM, AM-MSCCM, pAT-MSCCM, pAM-MSCCM) with the addition of 2% FBS and 2% ATB/ATM, followed by 5-day incubation in fresh MSCCM with 2% ATB/ATM. SH-SY5Y cells that were induced toward neuron-like differentiation by: (i) standard differentiation protocol via RA and BDNF and (ii) MSCCM (primed, non-primed) were processed for immunofluorescence analyses, neurite outgrowth and cell migration measurements.

### 2.12. Immunofluorescence

After being cultured on PureCol-coated coverslips, SH-SY5Y cells were rinsed in PBS, fixed in 4% paraformaldehyde, and then washed three times in PBS with 0.2% Triton X-100. After blocking with 10% normal goat serum in PBS for 120 minutes at room temperature (RT), cells were incubated with primary antibody (anti-beta III tubulin, Mouse monoclonal, ab78078, Abcam, United States) at 4°C overnight. The following day, coverslips were washed with PBS, then incubated in the dark for two hours at room temperature with a secondary antibody conjugated with a fluorescent probe (Goat-antimouse, Alexa Fluor 488, Invitrogen, United States). Cells were washed two times before being incubated in DAPI Fluoroshield solution for 5 min. Cells were imaged using a Zeiss AxioVision fluorescence microscope.

### 2.13. Neurite outgrowth measurement

Using a Zeiss Axiovert 200 inverted microscope with a 20X objective, computer-assisted microscopy was used to capture images of cell cultures for the characteristics of the differentiated cells. Under a phase-contrast microscope, at least 50 cells from five randomly selected fields of each experimental condition were examined to test neurite outgrowth (n = 50). The average total neurite length and average neurite count were then determined. The corresponding neurite length in cultures was analyzed using a conventional neurite-tracing procedure and by manually tracing the length of neurites using a software package ‘Draw a measurement shape’ (Zen Core v3.1). For neurite number estimation, we manually counted the number of cells (‘Number of events’) and traced the corresponding neurites coming from the cells, and calculated the average number of neurites per cell.

### 2.14. Scratch test

The wound healing assay was used to test SH-SY5Y cell migration. Cells were plated in a 24-well plate at 20×10^3^cells/well density in DMEM HG containing 10% FBS and 2% ATB/ATM. Once the cells were confluent, a 200 μL sterile plastic tip was used to make a scratch through the cell monolayer. Plates were then washed with PBS to remove non-adherent cells. Cells were cultured in conditioned media (AM-MSCCM, pAM-MSCCM, AT-MSCCM, pAT-MSCCM supplemented with 2% ATB/ATM) for 72 h. Cells cultured in DMEM HG with ATB/ATM were used as control. The cell-free area was observed under an inverted microscope and images were captured at 0, 24, 48, and 72 h after wounding using microscopy (Zeiss Axiovert 200) equipped with a digital acquisition system. In the cell migration assay, the edges of the wounds were marked, and the areas of the wounds were calculated using an online, open-access software ImageJ Fiji Wound Healing Tool.

## 3. Results

### 3.1. Morphology, immunophenotype and trilineage differentiation of AT-MSC and AM-MSC

**Figure 2:**
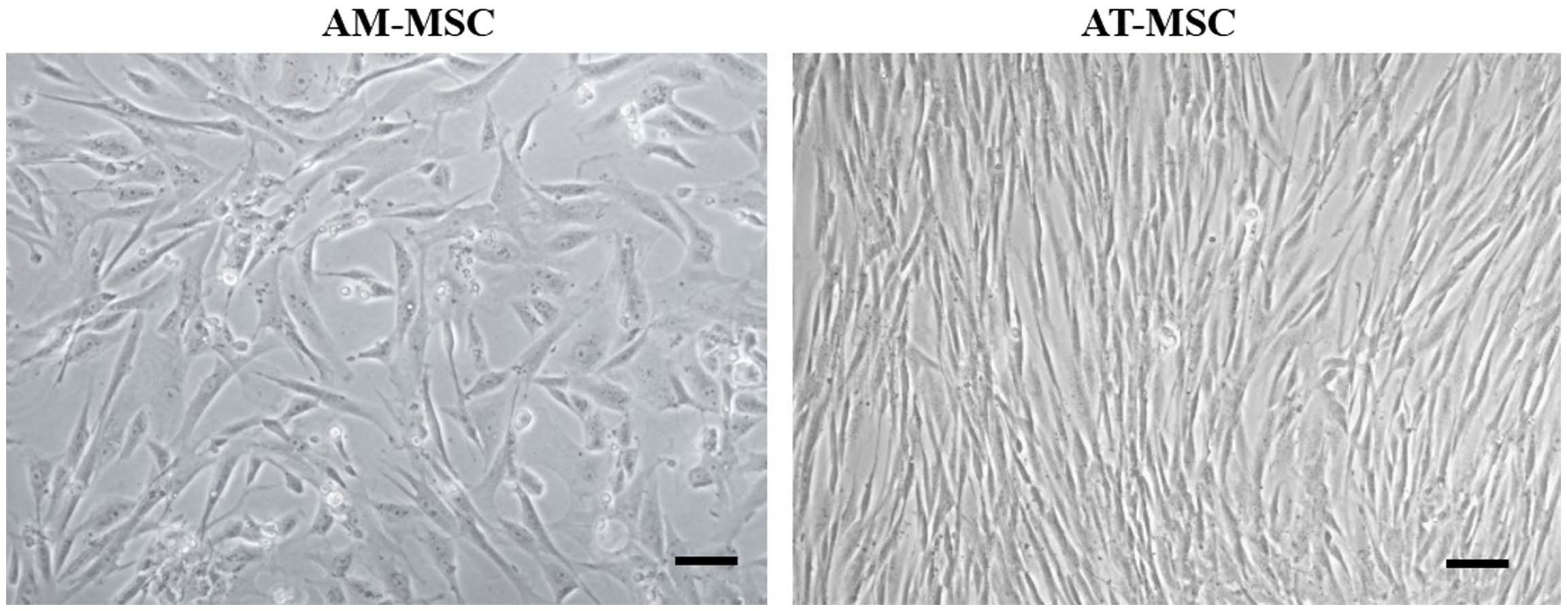
Morphology of canine AM-MSC and AT-MSC. AM-MSC exhibited fibroblast-like morphology, while AT-MSC were spindle-shaped, both typical for mesenchymal stromal perivascular cells. Scale bar 50 μm.

Canine AM-MSC and AT-MSC in passage 3 (P3) were identified based on morphology, immunophenotype, and differentiation potential. The cells harvested from canine perinatal (amnion) and adult (adipose tissue) tissues were adherent to a plastic surface and showed the spindle-shaped morphology typical for mesenchymal cells (Figure 2).

**Figure 3:**
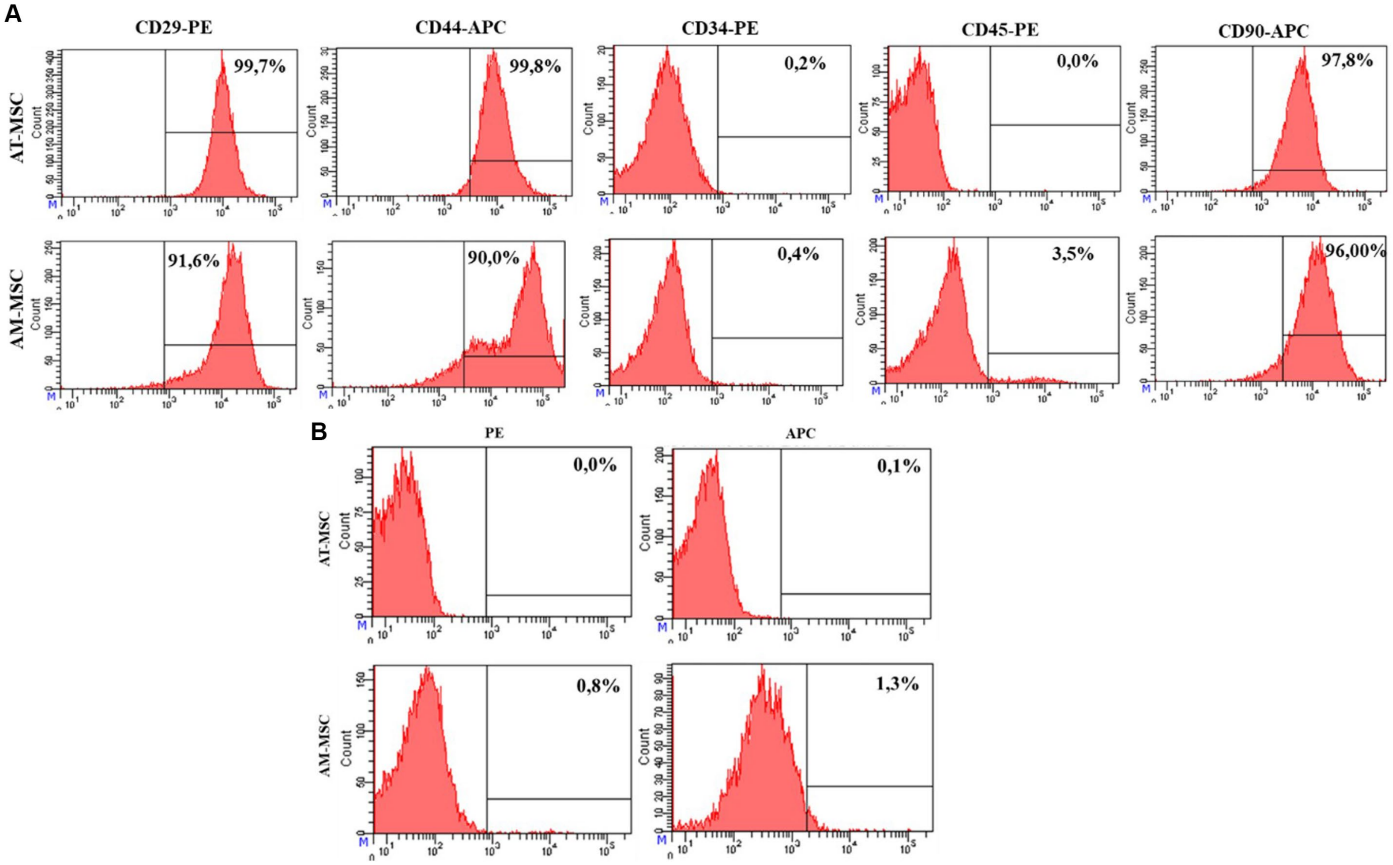
FACS analysis of the expression of the AT-MSC and AM-MSC surface markers at passage 3. **(A)** AT-MSC and AM-MSC expressed typical mesenchymal markers CD29, CD44, and CD90 but not CD34 and CD45. **(B)** The negative control is represented by unlabeled cells to exclude autofluorescence.

AM-MSC and AT-MSC expressed CD29 (AT-MSC 99.7%, AM-MSC 91.6%), CD44 (AT-MSC 99.8%, AM-MSC 90.0%), and CD90 (AT-MSC 97.8%, AM-MSC 96.0%), cells were negative for CD34 (AT-MSC 0.2%, AM-MSC 0.4%) and CD45 (AT-MSC 0.0%, AM-MSC 3.5%) (Figure 3).

**Figure 4:**
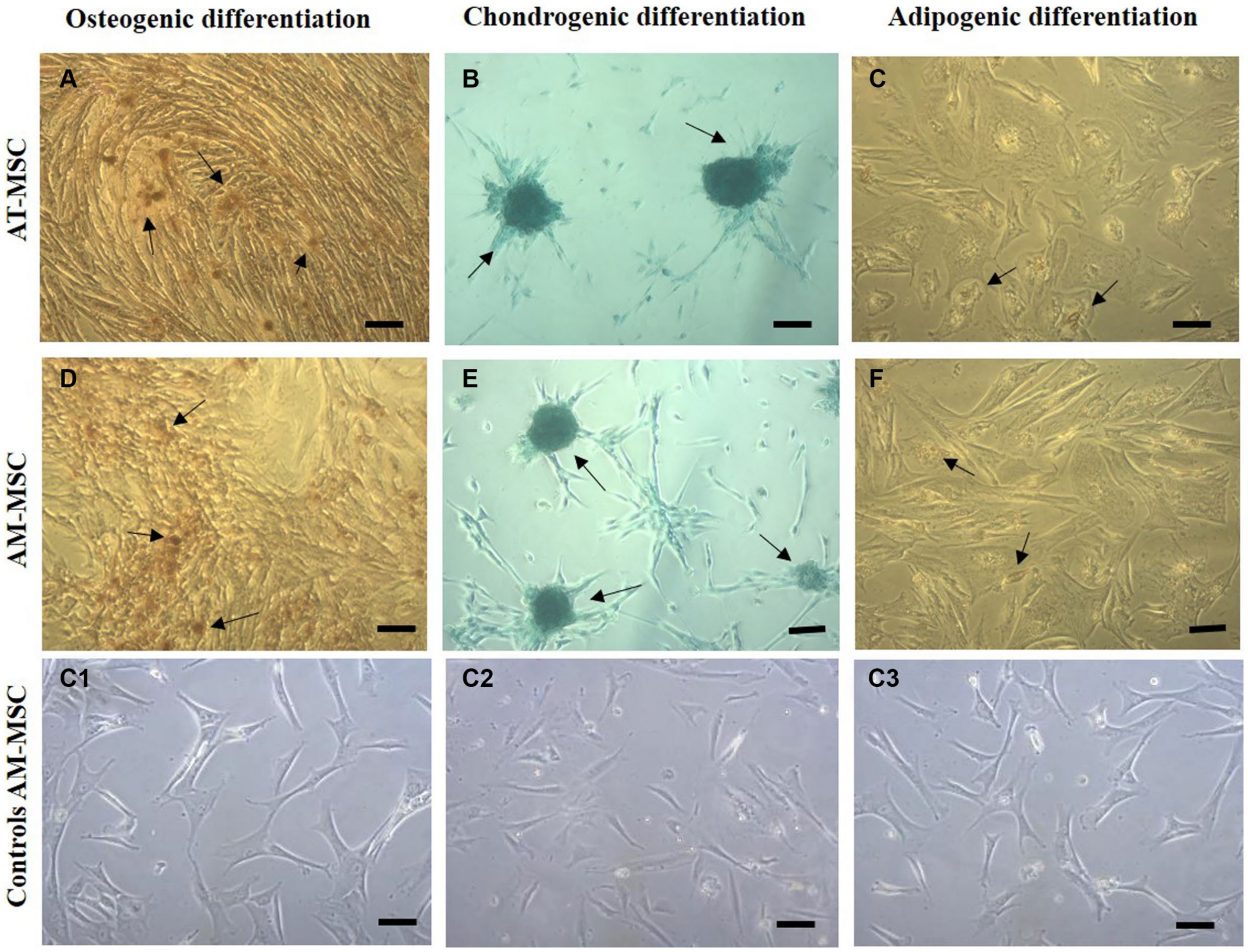
Multilineage differentiation of cells in passage 3. Canine AT-MSC and AM-MSC successfully underwent osteogenic **(A,D)**, chondrogenic **(B,E)**, and weaker adipogenic differentiation **(C,F)**. Respective negative controls **(C1,C2,C3)** are shown as AM-MSC cells where no differentiation media was used.

The MSC were subjected to osteogenic, chondrogenic, and adipogenic differentiation in commercial differentiation media (Figure 4). Alizarin red staining demonstrated that calcium deposits (indicated by arrows) were formed in MSC after 3 weeks of osteogenic induction (Figure 4A). After 21 days of chondrogenic induction, cells were stained with Alcian blue, positive (blue) acidic proteoglycans (arrows) indicating the formation of chondrocyte-like cells (Figure 4B). Intracellular Oil red staining showed a weak formation of lipid vacuoles (shown by arrows) typical of adipocytes after 3 weeks of adipogenic induction (Figure 4C).

### 3.2. Secretory profile of MSCCM

**Figure 5:**
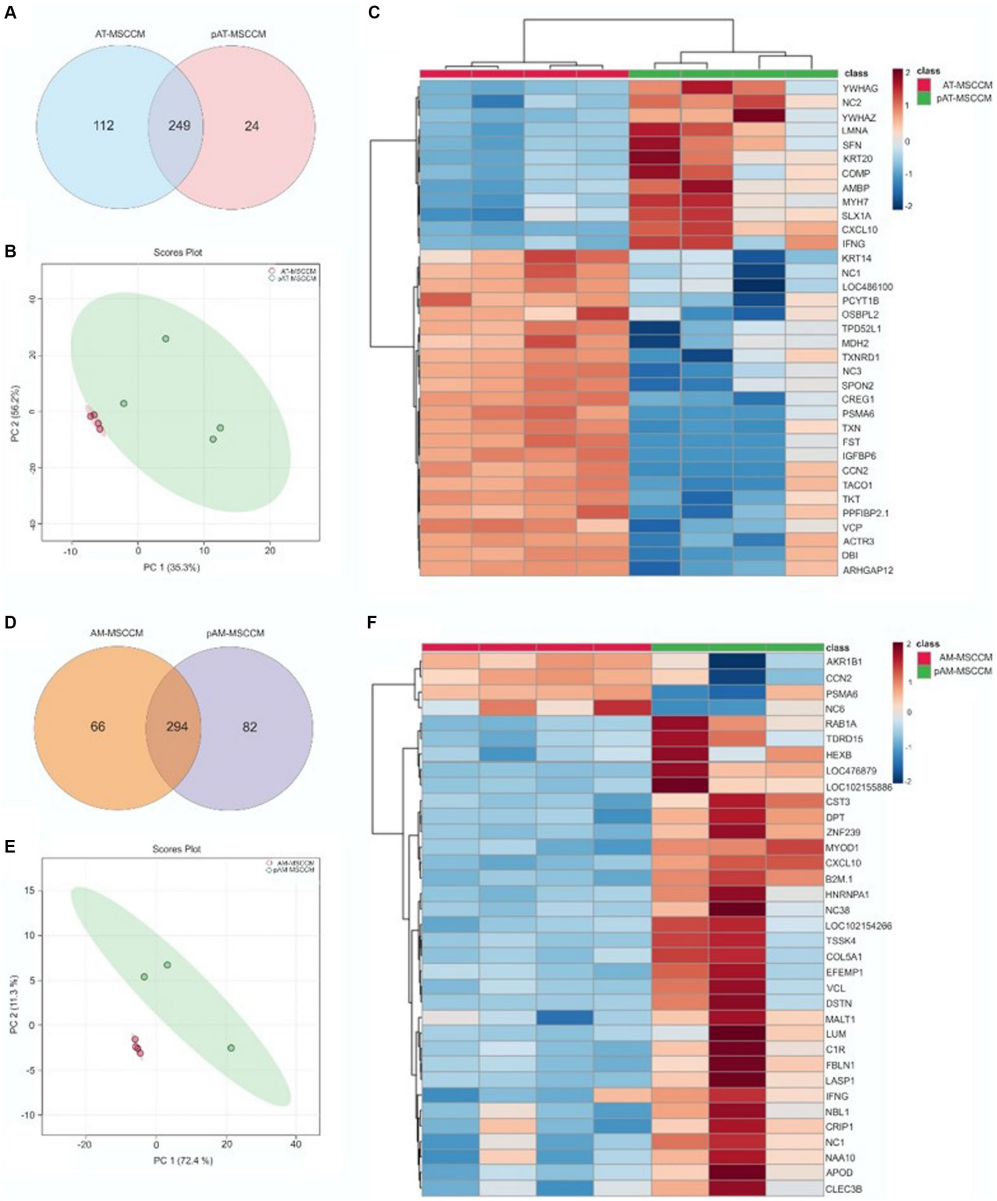
LC/MS–MS analysis of MSCCM and pMSCCM. **(A,D)** Venn diagrams representing the total amount of identified proteins in MSCCM and pMSCCM. **(B,E)** Principal component analysis scores plot showing clustering of MSCCM and pMSCCM. **(C,F)** Heat maps of differentially expressed proteins Red and blue colors represent up-regulated and down-regulated proteins, respectively.

The proteomes of MSCCM and pMSCCM were analyzed based on the protein detection- expressions measured by LC/MS–MS [liquid chromatography (LC) tandem mass spectrometry (MS)]. A total of 585 different proteins were identified. The MSCCMs and pMSCCMs had different proteomic profiles (normalized heat maps Figures 5C, F). For adipose tissue derived-MSCCM, 249 proteins were common to the AT-MSCCM and pAT-MSCCM, with 112 proteins being exclusive to AT-MSCCM and 24 proteins found only in the pAT-MSCCM group (Figure 5A). For CMs derived from AM-MSC, we found 294 proteins common to both AM-MSCCM and pAM-MSCCM. There were 66 proteins exclusive to AM-MSCCM, and 82 to pAM-MSCCM (Figure 5D). The overall highest number of proteins were found in AT-MSCCM and pAM-MSCCM.

Proteins involved in neuroregeneration, proliferation, cell migration, and neurite production were given special attention. In our conditioned media, we identified neuroprotective Insulin-like growth factor II (IGF-II), Connective tissue growth factor (CTGF), and Neuritin. All of them have been associated with promoting neurite growth (Zhang et al., 2017). Furthermore, we have detected Angiopoietin-like 4, which supported neurogenesis in an ischemic stroke rodent model (Qiu et al., 2021), Decorin, which supported axon growth in the SCI model (Biglari et al., 2012), as well as CSF1 (Zhang et al., 2017). Other detected factors associated with axon repair include Profilin 1, eukaryotic elongation factor 1 alpha (eEF1A) and also Neuroserpin, a regulator of axogenesis (Pinto-Costa et al., 2020; Godinez et al., 2022; Romaus-Sanjurjo et al., 2022).

### 3.3. Anti-inflammatory effect of MSCCM

**Figure 6:**
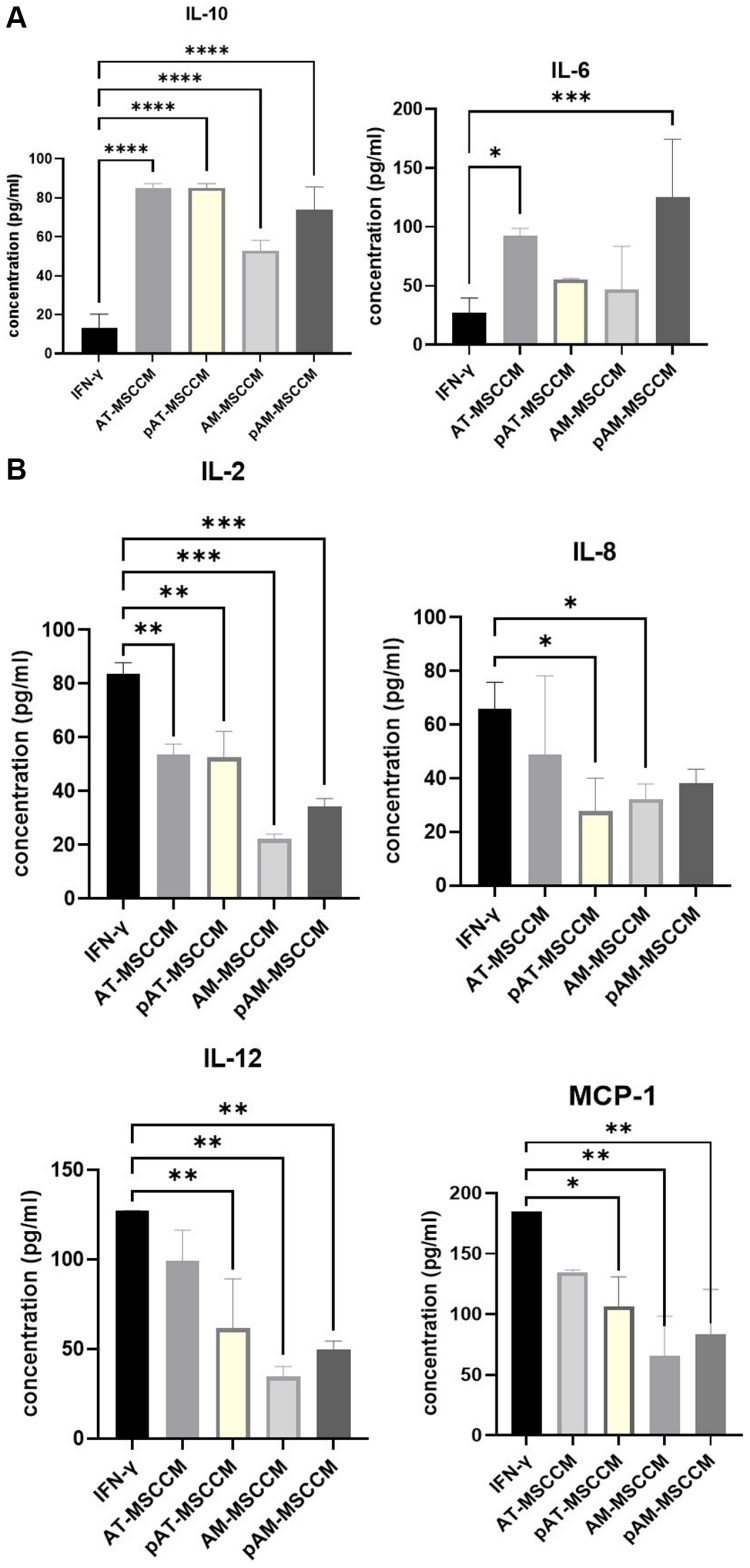
**(A)** Graphs showing changes in amounts of anti-inflammatory cytokines IL-10 and IL-6 (both pro and anti-inflammatory properties) detected by Luminex analyses. **(B)** Graphs showing changes in amounts of pro-inflammatory cytokines IL-2, IL-8, IL-12, and MCP-1 detected by Luminex analyses. Analytes released by SH-SY5Y cells after 24-h induction with IFN-γ and SH-SY5Y cultured in IFN-γ and with subsequent treatment with MSCCM for 24 h were measured. ANOVA Dunett’s multiple comparisons tests *p < 0.05, **p < 0.01, ***p < 0.001, ****p < 0.0001.

One of the aims of this study was to assess the effects of MSCCM on secretion profiles of cytokines, chemokines, and growth factors from cell line SH-SY5Y. Firstly, as expected pre-incubation of SH-SY5Y for 24 h with IFN-γ increased the output of pro-inflammatory cytokines IL-2, IL-6, IL-12/IL-23p40, chemokine IL-8 and monocyte chemoattractant protein 1 (MCP-1), on the other hand, the anti-inflammatory cytokine IL-10 output was decreased. Therefore, we considered this group as an IFN-γ group and studied whether additional treatment with MSCCM could reduce the value of pro-inflammatory and increase the value of anti-inflammatory cytokines/chemokines. Overall MSCCM significantly (*p < 0.05) or partially increased IL-10, IL-6 but decreased IL-2, IL-8, IL12 and MCP-1 output (Figures 6A,B). Interestingly, incubation with all MSCCM (AT-MSCCM, pAT-MSCCM, AM-MSCCM, pAM-MSCCM) significantly increased IL-10 (****p < 0.0001) with the highest increase detected in AT-MSCCM and pAT-MSCCM samples. For IL-6 a cytokine that can have both pro-inflammatory and anti-inflammatory properties depending on the context and concentration, pAM-MSCCM significantly increased IL-6 (***p < 0.001), followed by AT-MSCCM (*p < 0.05) while only a partial increase in groups of AM-MSCCM and pAT-MSCCM was detected. As for the concentration of proinflammatory mediators, we noticed a significant decrease in the concentration of cytokines IL-2 [significant decrease in AT-MSCCM (**p < 0.01), pAT-MSCCM (**), with the highest decrease in AM-MSCCM (***) followed by pAM-MSCCM (***)], IL-8 [significant decrease in pATMSCCM (*) and AM-MSCCM (*)], IL-12 [significant decrease in AM-MSCCM (**), followed by pAM-MSCCM (**) and pAT-MSCCM (**)] and MCP-1 [significant decrease in AM-MSCCM (**), followed by pAM-MSCCM (**) and pAT-MSCCM (*)] compared to the IFN-γ group (Figure 6B). Our results demonstrate that conditioned media from MSC attenuates IFN-γ-induced inflammatory responses by SH-SY5Y cells, suggesting their potential role in ameliorating neuroinflammation.

### 3.4. The effect of conditioned media on proliferation and metabolic activity

**Figure 7:**
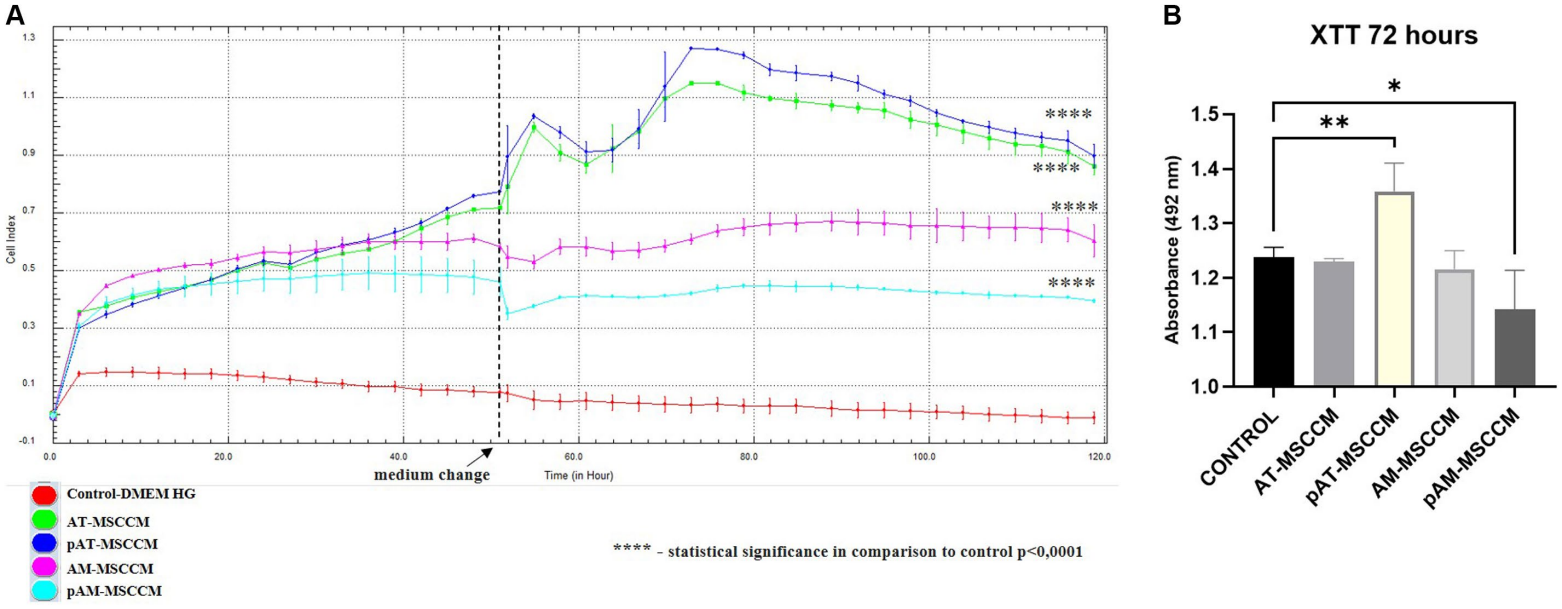
The effect of conditioned media on proliferation and metabolic activity. **(A)** Time-dependent proliferation profile of SH-SY5Y cultured in MSCCM. Proliferation curves of SH-SY5Y cells as generated by xCELLigence RTCA during 120 h. **(B)** Cell proliferation and metabolic activity were determined by the XTT assay after 72 h of incubation in DMEM medium supplemented with ATB/ATM and for experimental groups in conditioned media (AT-MSCCM, pAT-MSCCM, AM-MSCCM, pAM-MSCCM). *p < 0.05, **p < 0.01, ***p < 0.001, ****p < 0.0001. In both cases GraphPad Prism software was used for statistical analysis, utilizing the ANOVA Dunett’s multiple comparisons tests (*p < 0.05, **p < 0.01, ***p < 0.001, ****p < 0.0001).

To measure the influence of all MSCCM on the proliferation of SH-SY5Y cells by RTCA, the cells were treated with all samples of MSCCM (AT–MSCCM, AM–MSCCM, pAT–MSCCM, pAM–MSCCM) and control medium (DMEM with the ATB/ATM) and were monitored by RTCA for 120 h, with the media changed twice during the experiment. The results of ANOVA are displayed in Figure 7A. We observed a significantly (****p < 0.0001) increased adherence of SH-SY5Y cells in all experimental groups treated with conditioned media compared to the DMEM control (red line), represented by SH-SY5Y cells cultured in the control DMEM medium, from the second hour until the end of 120-h analysis. The AT-MSCCM and pAT-MSCCM groups had the highest adherence. The differences in adherence between the SH-SY5Y cells treated with conditioned media obtained from adult and perinatal tissue (AT vs. AM) were not significant. Moreover, no differences between primed and unprimed MSCCM were observed, although the pAT-MSCCM group had the highest adherence. These data demonstrate that the growth curve of the cells following MSCCM treatment was significantly higher (*p* < 0.0001) than in the control group given that Cell Index (CI) values are related to the number of cells, the results indicate a positive effect of MSCCM on proliferation.

To confirm the positive effects of tested MSCCM on metabolic activity, a standard colorimetric XTT assay was used simultaneously with RTCA where cells from the same passage were used. XTT is based on the analysis of mitochondrial metabolic activity in cells that have been exposed to the test substance (in this case MSCCM). The results of the XTT test, carried out on SH-SY5Y cells, after 72 h of exposure to MSCCM, are shown in Figure 7B. Interestingly, based on ANOVA results, we observed a significant increase (**p < 0.01) in metabolic activity only in the pAT-MSCCM group, with no significance measured between groups AT-MSCCM and AM-MSCCM nor between groups primed and not primed with IFN-γ. The coefficient value of Pearson’s correlation test between RTCA and XTT results was 0.864342, indicating a strong correlation.

### 3.5. The effect of conditioned media on differentiation and neurite outgrowth

Motor and sensory functions are frequently lost as a result of axonal injury to the central nervous system. To boost the regeneration of severed axons, growth-inhibitory influences from the tissue environment must be neutralized, and the intrinsic growth potential of neurons must be stimulated. We looked into whether adding MSCCM to the neuroblastoma cell line SH-SY5Y could improve neurite outgrowth and differentiation. In a variety of biomedical applications, such as neurite outgrowth and differentiation assays (Hoffmann et al., 2020; Godinez et al., 2022) SH-SY5Y cell line is differentiated by retinoic acid treatment. One of the distinctive features of SH-SY5Y neuroblastoma cells is the ability for differentiation into cells with neuron-like morphology (Gao et al., 2010).

**Figure 8:**
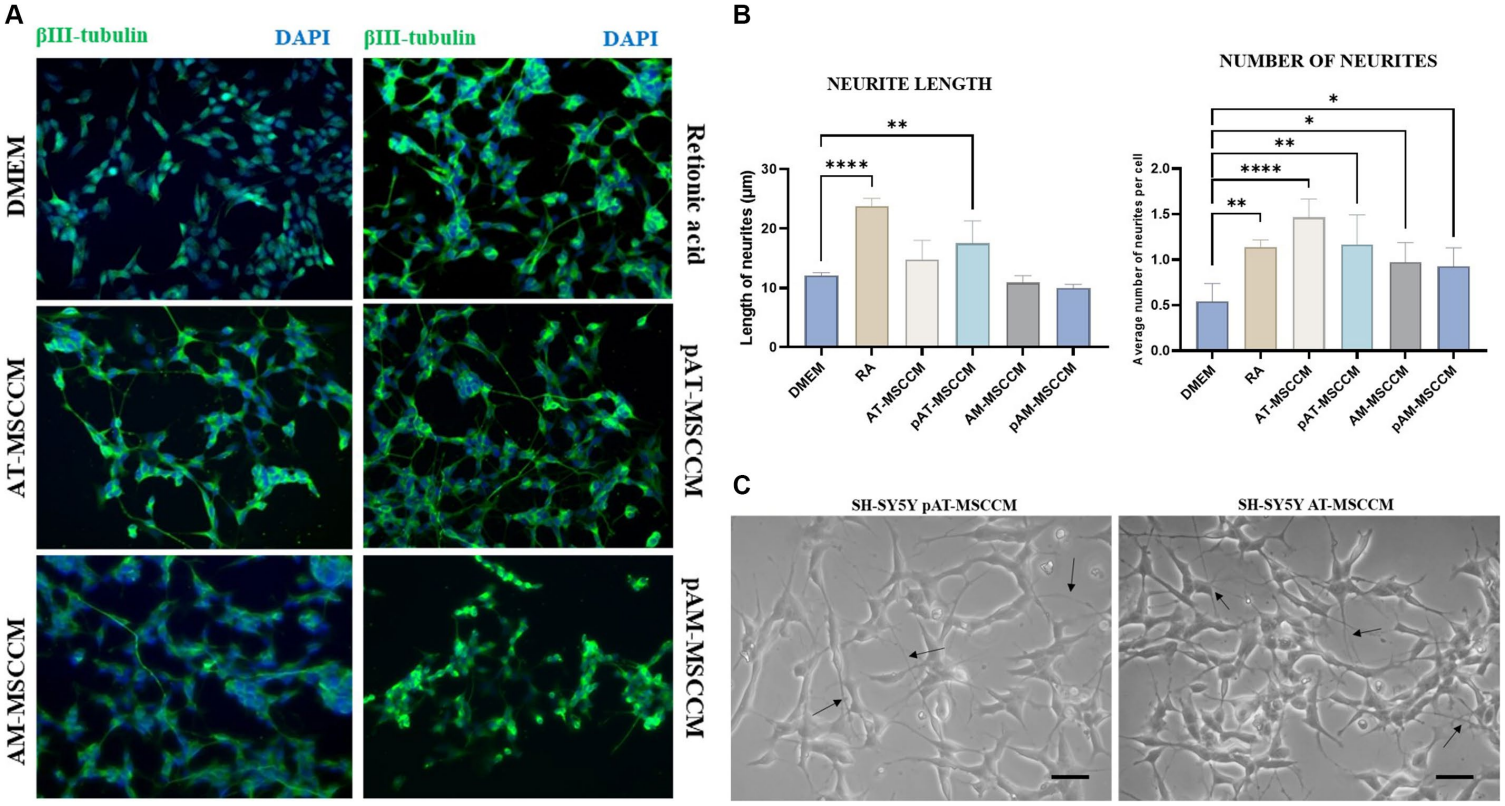
Effect of conditioned media on SH-SY5Y differentiation and neurite outgrowth. **(A)** Representative immunofluorescence images of β- III- tubulin positive cells (green) in differentiation media (Retinoic acid) and conditioned media (AT-MSCCM, pAT-MSCCM, AM-MSCCM, pAM-MSCCM) treatment groups. **(B)** Quantitative analyses of the average length of neurites and the average number of neurites per cell in different groups (ANOVA Dunett’s multiple comparisons tests * indicates *p* < 0.05). **(C)** Representative images of SH-SY5Y after culture in MSCCM for 12 days, note the long outgrowths in the group cultured in pAT-MSCCM (indicated by arrows) and the high number of neurites per cell in SH-SY5Y treated with AT-MSCCM (indicated by arrows). Scale bar: 20 μm.

To examine the role of MSCCM in differentiation, we compared the SH-SY5Y cells treated with all-trans retinoic acid (RA) and BDNF, serving as standard differentiation procedure with experimental differentiation processed by incubation SH-SY5Y cells with different MSCCM (AT-MSCCM, AM-MSCCM, pAT-MSCCM, pAM-MSCCM). After RA and MSCCM treatment for 12 days, the cells underwent morphological differentiation, revealing neuron-like characteristics and formation of long neurites (Figure 8A). The neuron-like differentiation was confirmed immunocytochemically by staining with beta-III tubulin, a typical marker for early neurons. Furthermore, the cultivation of SH-SY5Y cells in all conditioned media resulted in the differentiation of SH-SY5Y to neuron-like cells, positive for beta-III tubulin (Figure 8A).

We quantitatively evaluated the effects of MSCCM on neuronal outgrowth by determining the average neurite lengths and the average number of neurites per cell in groups of MSCCM, DMEM control group (SH-SY5Y cultured in DMEM), and RA differentiation group (SH-SY5Y cultured in DMEM with RA). ANOVA Dunett’s multiple comparisons test was used to evaluated the results as indicated in Figure 8B. Regarding the length of the neurites, we recorded longer neurites in the AT-MSCCM and pAT-MSCCM groups, but only the pAT-MSCCM group showed statistical significance (**p < 0.01) compared to the DMEM (control). On the other hand, when assessing neurite-promoting activity, we also focused on whether MSCCM influences neurite formation (average number of neurites per cell). Our data show that treating SH-SY5Y with all MSCCM resulted in a significantly higher number of neurites (**p < 0.01 for pAT-MSCCM, *p < 0.05 for AM-MSCCM and pAM-MSCCM), with the most neurites per cell observed in the AT-MSCCM group (****p < 0.0001), even more than in the control RA (differentiation group, **p < 0.01) (Figure 8B). We did not observe statistical significance between the IFN-γ-stimulated and non-stimulated groups. Overall, these results indicate the differentiation potential of MSCCM to form neuron-like cells as well as neurite-promoting activity based on the length and formation of neurites.

### 3.6. The effect of conditioned media on cell migration and re-growth

**Figure 9:**
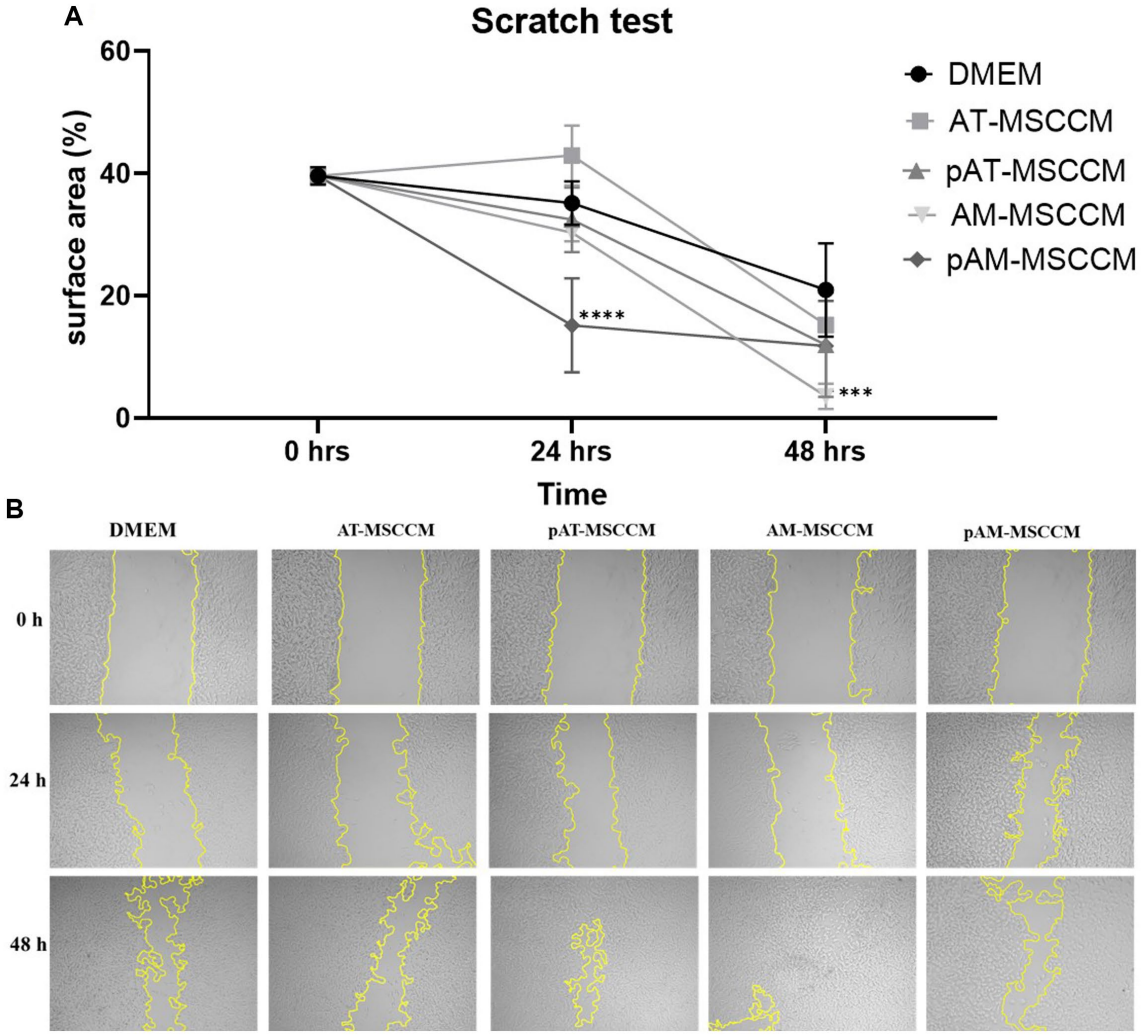
Cell migration detected by the scratch test (magnification, × 5). **(A)** Shows the statistical analysis of the separation distance of cells in all groups (percentage of empty area per field in all groups) by ANOVA (Dunett’s multiple comparisons tests, ***p < 0.001, ****p < 0.0001). **(B)** Representative photos of wells where we performed the Scratch test followed by 48-h treatment in Control medium (DMEM with ATB/ATM) and conditioned media (AT-MSCCM, pAT-MSCCM, AM-MSCCM, pAM-MSCCM).

Neurite regeneration is heavily influenced by cell migration and subsequent remodeling (Wu et al., 2012). Using the Scratch test we wanted to provide evidence that MSCCM contains molecules and bioactive factors that enhance SH-SY5Y cell migration and neurite re-growth of SH-SY5Y. Our results showcased in Figure 9 reveal that the addition of CM has an effect in regulating neurite re-growth and cell migration. After quantifying the results, we noted a significantly increased (****p < 0.0001) expansion of overgrowing cells and their neurites in the empty area in the SH-SY5Y group cultured in pAM-MSCCM after 24 h. After 72 h, we noted significance (***p < 0.001) in the experimental group AM-MSCCM compared to the negative control (cells cultured in DMEM with the addition of ATB/ATM).

## 4. Discussion

Mesenchymal stem cells (MSC) have been found to have a neuroprotective effect in various preclinical studies and clinical trials (Joyce et al., 2010). This effect is primarily attributed to the ability of MSC to modulate the immune system and release neurotrophic factors that promote the survival and regeneration of neurons. Priming MSC during culture has been presented as a way to increase their biological activity or activate new functions while maintaining their substantial plasticity *in vitro* (Peltzer et al., 2020). TNFα, IFN-γ, IL1β as well as hypoxic conditions are only a few of the many primings that have been researched (Andreeva et al., 2018). IFN-γ-preconditioning, in particular, has been the subject of extensive research and has been shown to increase the effectiveness of MSC and MSC-derived conditioned medium (MSCCM), in particular, by secreted molecular cargo of extracellular vesicles (EV), leading to improved repair of tissues (Peltzer et al., 2020).

The neurotrophic and immunomodulatory potential of conditioned media produced from adult (adipose tissue) and perinatal (amniotic membrane) MSC was characterized in the current study. We also studied the potential of stimulating the production of neurotropic and immunomodulatory bioactive molecules by priming MSC with an inflammatory cytokine IFN-γ.

Firstly we confirmed that both sources of cells (AT-MSC and AM-MSC) have been able to meet the criteria for MSC. They share ability to adhere to plastic surfaces, express certain cell surface markers, and differentiate into multiple cell types under specific *in vitro* conditions (Marion and Mao, 2006). The capacity of MSC isolated from both adipose tissue and the amniotic membrane to differentiate into osteogenic and chondrogenic lines was very good, whereas the ability to differentiate into adipogenic cells was very weak. Several studies claim that certain characteristics of MSC vary depending on the tissue source, mostly in terms of their ability to differentiate into different lineages, which partially agrees with our findings (Phinney and Sensebé, 2013). Overall our complex characterization validates the potential use of both sources in various MSC-cell-based and cell-free applications. These results are consistent with previous studies of AT-MSC and AM-MSC, indicating the mesenchymal origin of isolated MSC cells (Kern et al., 2006).

MSC mediate their function in a variety of ways, including the secretion of biologically active molecules and trophic factors thus provoking neuroprotection and neurogenesis (Yari et al., 2022). Using LC/MS–MS analysis, we detected 585 different proteins in a total of all conditioned media groups, 361 proteins in AT-MSCCM, 273 proteins in pAT-MSCCM, 360 in AM-MSCCM, and 376 proteins in pAM-MSCCM. Overall, conditioned media from perinatal sources (AM-MSCCM, pAM-MSCCM) contained more proteins than conditioned media from adult adipose tissue sources (AT-MSCCM, pAT-MSCCM), as confirmed by the authors who compared the proteomic composition of human secretomes from adult and fetal source MSC which correlates with our data (Shin et al., 2021). Although there were differences in the amount of protein contents between AT-MSCCM and AM- MSCCM primed or non-primed, the most important is their qualitative properties and their functionality toward neuroregeneration and neuroprotection.

In terms of the identification of specific proteins linked to neuroregeneration, neurotrophicy, and their influence on neurite proliferation and outgrowth, we managed to detect CTGF, which promotes axonal regeneration (Negro et al., 2022). We also found neuroprotective IGF-II, which increased sensory axon regeneration in rats (Glazner et al., 1993), along with eEF1A proteins that promote corticospinal axon repair after injury (Romaus-Sanjurjo et al., 2022). The conditioned media also contained Neuritin, which via Notch Signaling inhibition promotes neurite growth (Zhang et al., 2017), Angiopoietin-like 4, known to promote neurogenesis in a mouse rodent model with acute ischemic stroke (Qiu et al., 2021; Liu et al., 2022), Profilin 1, identified as an important regulator of axonal regeneration (Pinto-Costa et al., 2020), and Neuroserpin, a crucial regulator for axogenesis, synaptic modeling, and cell–cell interactions (Godinez et al., 2022). The detection of Decorin, which is linked to promoting nerve axon growth in cultured dorsal root ganglia and after spinal cord injury *in vivo*, was also interesting, and in the cultured dorsal root ganglia (Wu et al., 2020) and also Macrophage colony-stimulating factor1 (CSF1), which helps to maintain microglial roles of synaptic pruning, the release of neurotrophic factors, and promotion of brain connectivity (Bo and Bo, 2021). Other researchers employing cytokine array techniques also found brain-derived growth factor (BDNF), glial-derived growth factor (GDNF), nerve growth factor (NGF), NT-3, and basic fibroblast growth factor (bFGF) in MSC secretomes (Cofano et al., 2019; Petrenko et al., 2020). MSC may stimulate neurogenesis, axonal growth, re-myelination, and cell metabolism through the action of these factors, which can also prevent nerve degeneration (Numakawa and Kajihara, 2023).

Inflammatory processes in the nervous system can lead to tissue damage, neuronal death, and impaired neurological function (Skaper et al., 2018). Suppressing the inflammatory response is an important therapeutic strategy in the treatment of these diseases. Therefore, we examined the immunomodulatory impact of all MSCCM (AT-MSCCM, AM-MSCCM with and without priming by IFN-γ) on SH-SY5Y cell line by evaluating the expression of selected pro and anti-inflammatory markers on the Luminex assay. The highest increases in IL-10 were seen when SH-SY5Y were treated with AT-MSCCM and pAT-MSCCM. IL-10 can help to counteract inflammatory processes by inhibiting the production of pro-inflammatory cytokines and promoting the expression of anti-inflammatory factors. This can lead to a reduction in inflammation and tissue damage, allowing for the initiation of the repair and regeneration process. Furthermore, IL-10 has been shown to promote neurogenesis (generating new neurons in the brain), enhance the survival and function of neurons, and promote the growth and regeneration of nerve fibers (Perez-Asensio et al., 2013; Chen et al., 2016). Overall, the findings show that MSC-conditioned media reduces IFN-γ -induced inflammatory responses in SH-SY5Y cells (decrease of IL-2, IL-8, IL-12, MCP-1), indicating that it may have a role in reducing neuroinflammation. As for the pro-inflammatory cytokines, IL-2 is a pleiotropic cytokine that promotes T-cell expansion, increases NK cytolytic activity, triggers regulatory T cell differentiation, and facilitates activation-induced cell death. According to current research, IL-2 also disrupts the blood–brain barrier and changes brain microcirculation (Liao et al., 2011; Wylezinski and Hawiger, 2016). Multiple neurodegenerative illnesses have been linked to the chemokine IL-8. In the brain, increased IL-8 levels in response to injury draw neutrophils into the central nervous system (CNS), where they degranulate and produce chemoattractants for T cells (Robinson et al., 2020). IL-12, which is produced in the initial phases of inflammation in response to infection or other stimuli, helps the inflammation itself, mainly in a way of the activation of macrophages. IL-12 also helps to the synthesis of IFN-γ and other cytokines (Gee et al., 2009). Inhibiting MCP-1/CCR2 signaling, for instance, decreased ethanol-induced microglial activation/neuroinflammation and improved neurodegeneration in the developing brain (Zhang et al., 2018). This suggests that MCP-1/CCR2 signaling may be involved in neuroinflammatory conditions (Zhang et al., 2018). Regarding IL-6, a cytokine that is not only involved in inflammation and infection responses, but also in the regulation of metabolic, regenerative, and neural processes (Scheller et al., 2011, p. 6), significant increases in IL-6 were also observed with pAM-MSCCM and AT-MSCCM, but not with AM-MSCCM or pAT-MSCCM group.

We used functional analyses on the SH-SY5Y cell line, which is frequently used to study diseases of the nervous system (Parkinson’s disease in particular), to evaluate the effectiveness of the AT-MSCCM and AM-MSCCM and the functionality of factors and bioactive proteins they contain. One of our aims was to observe if there is an increase in the proliferation of the SH-SY5Y after conditioned media administration. We used the xCELLigence RTCA system and XTT assay to measure cell proliferation and the metabolic activity of cells in each group. Our findings show that all conditioned media increased adherence and proliferation of SH-SY5Y during the 120-h study when compared to the control group cultured in the DMEM medium. The pAT-MSCCM group had the highest adherence, followed by the AT-MSCCM group, and then by the AM-MSCCM and pAM-MSCCM groups. We also found a significant increase (*p* < 0,01) in metabolic activity in the pAT-MSCCM group when using the XTT test.

These findings correlate with our findings of proteomic analyses and other authors pointing to the positive impact of secretome derived from MSC on neural progenitor cell (NPC) proliferation (Teixeira et al., 2016). Particularly CTGF, which regulates the survival of interneurons, neuroprotective IGF-II, Decorin, which supports axon growth and neurotrophic Neuritin, which, in addition to axon regeneration, also promotes retinal ganglion cell survival (Minor et al., 2008; Khodosevich et al., 2013; Sharma et al., 2015; Beletskiy et al., 2021). Regarding which source of MSC has a higher proliferative capacity *per se*, authors have mixed opinions, while our previous study, comparing canine MSC, reported AT-MSC as cells with higher proliferation, according to Hass et al. comparing human MSC from neonatal and adult sources Hass et al. reported that neonatal-tissue-derived MSC has higher proliferative activity (Hass et al., 2011; Humenik et al., 2022). Our data are related to the findings of Wang et al., they demonstrated that MSC was able to regulate the survival, proliferation, and differentiation of neural stem cells through Notch signaling. The conditioned media also contained Neuritin, which via Notch Signaling inhibition promotes neurite growth (Zhang et al., 2017). In addition to controlling precursor cell fate, Notch activation is also involved in neuronal maturation, induction of neurite outgrowth, and prevention of apoptosis (Wang et al., 2009; Teixeira et al., 2016). According to Eleuteri and Fierabracci (2019). Additionally, MSC affect the activity of MAPK (mitogen-activated protein kinase), NOS (nitric oxide synthase), which generates NO (nitric oxide), that can inhibit the cell cycle via the JAK–STAT (Janus kinase/signal modulators and activators of transcription) pathway (Eleuteri and Fierabracci, 2019). The authors report that the MAPK signaling pathway is involved in the regulation of viability, cell proliferation, survival, and differentiation in embryonic development, and its role in neurodegenerative diseases is being studied (Jiao et al., 2017; Eleuteri and Fierabracci, 2019).

In terms of differentiation and growth, several studies have discovered that human and rodent MSC promote nerve cell survival and neurite extension *in vitro* (Hokari et al., 2008; Nakano et al., 2010; Nakamura et al., 2020). In our study, we discovered that all conditioned media, both stimulated and unstimulated with IFN-γ, promoted neuroblastoma line (Figure 8) differentiation, but there were some differences when we looked at neurite outgrowths. While we found significance in all conditioned media when counting the number of neurites per cell, with the highest number in AT-MSCCM and pAT-MSCCM, we found significance only in the pAT-MSCCM group when measuring the length of processes, indicating the superiority of the stimulated conditioned medium and the medium obtained from AT -MSC in terms of neurite-outgrowth promoting activity. Our results are in agreement with previous studies using rodent, primal, or human MSC to promote survival, neuronal differentiation, neurite outgrowth, and to protect motor-neuron-like NSC against scratch-injury-induced cell death (Lou et al., 2003; Rajan et al., 2017; Nakamura et al., 2020). Delfi et al. reported that *in vitro* differentiation as well as neurite outgrowth of SH-SY5Y was improved by canine MSCCM (Al Delfi et al., 2016). This ability of conditioned media can be attributed, in addition to the secretion of growth factors such as IGF II, Neuritin, and CTGF, found in the present study, and other factors like BDNF, GDNF, VEGF, to the modulation of signaling pathways such as phosphatidylinositol3-kinase/Akt signaling pathway and upregulation of the antiapoptotic Bcl-2 protein, cAMP-activated pathway, including PKA and PI3K (López-Carballo et al., 2002; Kovalevich and Langford, 2013). In the case of outgrowth, the erk1/2 signaling pathway and the SIRT1 signaling pathway must be highlighted (Liu et al., 2013; Khatib et al., 2019).

When we focused on the functional impact of primed and unprimed conditioned media on cell re-growth of SH-SY5Y, in this study we demonstrated that conditioned media treatment of SH-SY5Y regenerated neurites in the scratch assay. After 24 h, cells treated with primed pAM-MSCCM showed significant regrowth and regeneration of neurites compared to the control, as did cells treated with AM-MSCCM after 48 h (Figure 9). There was no significant effect on cell re-growth in the presence of AT-MSCCM or pAT-MSCCM compared to the control, indicating that these findings are consistent with previously reported results that MSC from neonatal sources appears to be the best cell source for healing wounds (Humenik et al., 2023). The effect of conditioned media on the re-growth of various cell lines has been described (Wang et al., 2022). The regulation of signaling cascades MAPK/ERK kinase (MEKK) kinase 1 (MEKK1) and ERK1/2 and JNK2 signaling appears to be critical for neuronal-like cell re-growth (Wu et al., 2012). Furthermore, MSC-Exosomes express CD73, an ecto 5-nucleotidase that converts AMP to adenosine before phosphorylating and activating survival kinases (Erk1/2 and Akt) (Colgan et al., 2006).

When we compare our findings to the neurotrophic functions of conditioned media derived from MSC from adult adipose tissue and neonatal amniotic tissue, we find that while AT-MSCCM has a greater effect on proliferation, metabolic activity, and differentiation- induced outgrowth, AM-MSCCM has a greater immunomodulatory capacity and influence on SH-SY5Y injury induced re-growth. These data should be considered as MSC source has been found to correlate with therapeutic efficacy. For instance, Zhou et al. showed that human AT-MSC xenotransplantation to rats with spinal cord injury increased angiogenesis and axonal regeneration in addition to higher functional recovery than BM-MSC (Zhou et al., 2013). The BDNF, VEGF, and HGF levels that AT-MSC produced were much higher. Although there were no differences between AT-MSC, BM-MSC, Wharton’s Jelly-Derived Mesenchymal Stem Cells (WJ-MSC), and Cord Blood-Derived Mesenchymal Stem Cells (CB-MSC), dogs who received MSC 1 week after spinal cord injury shown a significant improvement in functional recovery (Ryu et al., 2012).

Numerous primings, such as inflammatory (TNF, IFN-γ, IL1) or hypoxic conditions, have been studied to increase the therapeutic efficacy of MSC and MSC-derived conditioned medium (Andreeva et al., 2018). IFN-γ preconditioning has been the subject of extensive research. In our experiments, we observed a significant difference in immunomodulatory (changes in the values of IL-6 and IL-10) and anti-inflammatory activity (changes in the values of IL-2 and IL-8, IL-12) between the conditioned media that were and were not stimulated with IFN-γ. When compared to the control, primed pAT-MSCCM significantly increased neurite outgrowth in terms of SH-SY5Y outgrowth number and length. The authors’ views on priming differ considerably. According to Peltzer et al., interferon and hypoxia-priming have only little effects on the miRNA landscape of extracellular vesicles produced by human MSC (Sheng et al., 2008; Peltzer et al., 2020), despite Sheng *et al*’s assertion that immunosuppression of IFN-γ-primed MSC is crucial. These findings might open the door to the creation of a secure and efficient acellular therapy for the myriad neurological conditions that affect people. Although our results are very encouraging, using MSCCM in a clinical setting requires optimal and standardized manipulation, as well as a stronger comprehension of the neuroregenerative effect, immune biology, and interactions within the microenvironment.

## 5. Conclusion

In the present research, we thoroughly examined MSC-derived conditioned medium (CM), mainly for its neuroregeneration-promoting properties. Even though we found neurotrophic potential in all CM we confirmed the non-equality of CM isolated from MSC derived from different sources (canine adipose tissue, canine amniotic membrane tissue), regarding the content of proteins and bioactive factors, their ability to affect proliferation, metabolic activity, differentiation, neurite outgrowth, and re-growth of injured SH-SY5Y cells. We showed that AT-MSCCM was characterized by higher proliferative, metabolic activity-affecting capacity and the ability to impact neurite outgrowth (length and a number of neurites per cell). AM-MSCCM displayed more pronounced immunomodulatory activity and impact on migration and regrowth of injured SH-SY5Y compared to AT-MSCCM. Finally, priming with inflammatory cytokine (IFN-γ) induced proteomic profiles of CM as well as the enhanced impact on SH-SY5Y proliferation and neurite length when compared to non-primed CM from both sources. To expand the range of therapeutic characteristics and improve production techniques, further research must be done on MSCCM and its response to priming. Overall, our study presents critical data for the translation of fundamental MSC research to clinical manufacturing and therapeutic applications.

## Data availability statement

The original contributions presented in the study are included in the article/supplementary material, further inquiries can be directed to the corresponding author.

## Ethics statement

Ethical approval was not required for the studies on humans in accordance with the local legislation and institutional requirements because only commercially available established cell lines were used. The animal studies were approved by this study was approved by the institutional ethical committee of the University of Veterinary Medicine and Pharmacy in Kosice (EKY8/2021-02). The studies were conducted in accordance with the local legislation and institutional requirements. Written informed consent was obtained from the owners for the participation of their animals in this study.

## Author contributions

DC and NH: Conceptualization. NH, PM, LS, DM, DM, MM, PP, and FH: methodology. DM, DM, and PM: software. DC, NH, MM, and PP: writing – original draft preparation. DC, NH, PM, DM, and LS: writing – review and editing and funding acquisition. All authors contributed to the article and approved the submitted version.

## References

[ref1] Ahmadbeigi N. Shafiee A. Seyedjafari E. Gheisari Y. Vassei M. Amanpour S.. (2011). Early spontaneous immortalization and loss of plasticity of rabbit bone marrow mesenchymal stem cells. Cell Prolif. 44, 67–74. doi: 10.1111/j.1365-2184.2010.00731.x, PMID: 21199011 PMC6496252

[ref2] Al Delfi I. R. Sheard J. J. Wood C. R. Vernallis A. Innes J. F. Myint P.. (2016). Canine mesenchymal stem cells are neurotrophic and angiogenic: An in vitro assessment of their paracrine activity. Vet. J. 217, 10–17. doi: 10.1016/j.tvjl.2016.09.003 27810198

[ref3] Andreeva E. R. Udartseva O. O. Zhidkova O. V. Buravkov S. V. Ezdakova M. I. Buravkova L. B. (2018). IFN-gamma priming of adipose-derived stromal cells at “physiological” hypoxia. J. Cell. Physiol. 233, 1535–1547. doi: 10.1002/jcp.26046, PMID: 28600879

[ref4] Beletskiy A. Chesnokova E. Bal N. (2021). Insulin-like growth factor 2 as a possible neuroprotective agent and memory enhancer—its comparative expression, processing and signaling in mammalian CNS. Int. J. Mol. Sci. 22:1849. doi: 10.3390/ijms22041849, PMID: 33673334 PMC7918606

[ref5] Berebichez-Fridman R. Montero-Olvera P. R. (2018). Sources and clinical applications of mesenchymal stem cells. Sultan Qaboos Univ. Med. J. 18, e264–e277. doi: 10.18295/squmj.2018.18.03.002, PMID: 30607265 PMC6307657

[ref6] Biglari A. Salarinia R. Mazloomzadeh S. Jafari I. Ranjzad P. Kingston P. A. (2012). Repair of spinal cord injury (SCI) using monocytes transfected with adenoviral vector expressing Decorin in a rat SCI model. Mol. Therapy 20, 68–69

[ref7] Bo L. Bo X. (2021). Colony stimulating factor 1: friend or foe of neurons? Neural Regen. Res. 17, 773–774. doi: 10.4103/1673-5374.322451, PMID: 34472465 PMC8530122

[ref8] Chen H. Lin W. Zhang Y. Lin L. Chen J. Zeng Y.. (2016). IL-10 promotes neurite outgrowth and synapse formation in cultured cortical neurons after the oxygen-glucose deprivation via JAK1/STAT3 pathway. Sci. Rep. 6:30459. doi: 10.1038/srep30459, PMID: 27456198 PMC4960594

[ref9] Choudhery M. S. Badowski M. Muise A. Pierce J. Harris D. T. (2014). Donor age negatively impacts adipose tissue-derived mesenchymal stem cell expansion and differentiation. J. Transl. Med. 12:8. doi: 10.1186/1479-5876-12-8, PMID: 24397850 PMC3895760

[ref10] Coelho A. Alvites R. D. Branquinho M. V. Guerreiro S. G. Maurício A. C. (2020). Mesenchymal stem cells (MSCs) as a potential therapeutic strategy in COVID-19 patients: literature research. Front. Cell Dev. Biol. 8:602647. doi: 10.3389/fcell.2020.602647, PMID: 33330498 PMC7710935

[ref11] Cofano F. Boido M. Monticelli M. Zenga F. Ducati A. Vercelli A.. (2019). Mesenchymal stem cells for spinal cord injury: current options, limitations, and future of cell therapy. Int. J. Mol. Sci. 20:2698. doi: 10.3390/ijms20112698 31159345 PMC6600381

[ref12] Colgan S. P. Eltzschig H. K. Eckle T. Thompson L. F. (2006). Physiological roles for ecto-5′-nucleotidase (CD73). Purinergic Signal 2, 351–360. doi: 10.1007/s11302-005-5302-5, PMID: 18404475 PMC2254482

[ref14] Eleuteri S. Fierabracci A. (2019). Insights into the Secretome of mesenchymal stem cells and its potential applications. Int. J. Mol. Sci. 20:4597. doi: 10.3390/ijms20184597, PMID: 31533317 PMC6770239

[ref15] Gao X. Joselin A. P. Wang L. Kar A. Ray P. Bateman A.. (2010). Progranulin promotes neurite outgrowth and neuronal differentiation by regulating GSK-3β. Protein Cell 1, 552–562. doi: 10.1007/s13238-010-0067-1, PMID: 21204008 PMC4875315

[ref16] Gee K. Guzzo C. Che Mat N. F. Ma W. Kumar A. (2009). The IL-12 family of cytokines in infection, inflammation and autoimmune disorders. Inflamm. Allergy Drug Targets 8, 40–52. doi: 10.2174/187152809787582507 19275692

[ref17] Glazner G. W. Lupien S. Miller J. A. Ishii D. N. (1993). Insulin-like growth factor II increases the rate of sciatic nerve regeneration in rats. Neuroscience 54, 791–797. doi: 10.1016/0306-4522(93)90248-e, PMID: 8332262

[ref18] Godinez A. Rajput R. Chitranshi N. Gupta V. Basavarajappa D. Sharma S.. (2022). Neuroserpin, a crucial regulator for axogenesis, synaptic modelling and cell–cell interactions in the pathophysiology of neurological disease. Cell. Mol. Life Sci. 79:172. doi: 10.1007/s00018-022-04185-6, PMID: 35244780 PMC8897380

[ref19] Gugliandolo A. Mazzon E. (2022). Dental mesenchymal stem cell Secretome: An intriguing approach for neuroprotection and Neuroregeneration. Int. J. Mol. Sci. 23:456. doi: 10.3390/ijms23010456, PMID: 35008878 PMC8745761

[ref21] Hass R. Kasper C. Böhm S. Jacobs R. (2011). Different populations and sources of human mesenchymal stem cells (MSC): a comparison of adult and neonatal tissue-derived MSC. Cell Commun. Signal 9:12. doi: 10.1186/1478-811X-9-12, PMID: 21569606 PMC3117820

[ref23] Hoffmann M. Kleine-Weber H. Schroeder S. Krüger N. Herrler T. Erichsen S.. (2020). SARS-CoV-2 cell entry depends on ACE2 and TMPRSS2 and is blocked by a clinically proven protease inhibitor. Cells 181, 271–280.e8. doi: 10.1016/j.cell.2020.02.052 PMC7102627 32142651

[ref24] Hokari M. Kuroda S. Shichinohe H. Yano S. Hida K. Iwasaki Y. (2008). Bone marrow stromal cells protect and repair damaged neurons through multiple mechanisms. J. Neurosci. Res. 86, 1024–1035. doi: 10.1002/jnr.21572, PMID: 18030676

[ref25] Humenik F. Maloveska M. Hudakova N. Petrouskova P. Hornakova L. Domaniza M.. (2022). A comparative study of canine mesenchymal stem cells isolated from different sources. Animals 12:1502. doi: 10.3390/ani12121502, PMID: 35739839 PMC9219547

[ref26] Humenik F. Maloveská M. Hudáková N. Petroušková P. Šufliarska Z. Horňáková Ľ.. (2023). Impact of canine amniotic mesenchymal stem cell conditioned media on the wound healing process: in vitro and in vivo study. Int. J. Mol. Sci. 24:8214. doi: 10.3390/ijms24098214, PMID: 37175924 PMC10179513

[ref28] Jiao Q. Li X. An J. Zhang Z. Chen X. Tan J.. (2017). Cell-cell connection enhances proliferation and neuronal differentiation of rat embryonic neural stem/progenitor cells. Front. Cell. Neurosci. 11:200. doi: 10.3389/fncel.2017.00200, PMID: 28785204 PMC5519523

[ref29] Jovic D. Yu Y. Wang D. Wang K. Li H. Xu F.. (2022). A brief overview of global trends in MSC-based cell therapy. Stem Cell Rev and Rep 18, 1525–1545. doi: 10.1007/s12015-022-10369-1, PMID: 35344199 PMC8958818

[ref30] Joyce N. Annett G. Wirthlin L. Olson S. Bauer G. Nolta J. A. (2010). Mesenchymal stem cells for the treatment of neurodegenerative disease. Regen. Med. 5, 933–946. doi: 10.2217/rme.10.72, PMID: 21082892 PMC3017479

[ref31] Kern S. Eichler H. Stoeve J. Klüter H. Bieback K. (2006). Comparative analysis of mesenchymal stem cells from bone marrow, umbilical cord blood, or adipose tissue. Stem Cells 24, 1294–1301. doi: 10.1634/stemcells.2005-0342 16410387

[ref32] Khatib T. Marini P. Nunna S. Chisholm D. R. Whiting A. Redfern C.. (2019). Genomic and non-genomic pathways are both crucial for peak induction of neurite outgrowth by retinoids. Cell Commun. Signal 17:40. doi: 10.1186/s12964-019-0352-4, PMID: 31046795 PMC6498645

[ref33] Khodosevich K. Lazarini F. von Engelhardt J. Kaneko H. Lledo P.-M. Monyer H. (2013). Connective tissue growth factor regulates interneuron survival and information processing in the olfactory bulb. Neuron 79, 1136–1151. doi: 10.1016/j.neuron.2013.07.011, PMID: 23993699

[ref34] Kovalevich J. Langford D. (2013). Considerations for the use of SH-SY5Y neuroblastoma cells in neurobiology. Methods Mol. Biol. 1078, 9–21. doi: 10.1007/978-1-62703-640-5_2, PMID: 23975817 PMC5127451

[ref35] Kvistad C. E. Kråkenes T. Gjerde C. Mustafa K. Rekand T. Bø L. (2022). Safety and clinical efficacy of mesenchymal stem cell treatment in traumatic spinal cord injury, multiple sclerosis and ischemic stroke – a systematic review and Meta-analysis. Front. Neurol. 13:891514. doi: 10.3389/fneur.2022.891514, PMID: 35711260 PMC9196044

[ref36] Liao W. Lin J.-X. Leonard W. J. (2011). IL-2 family cytokines: new insights into the complex roles of IL-2 as a broad regulator of T helper cell differentiation. Curr. Opin. Immunol. 23, 598–604. doi: 10.1016/j.coi.2011.08.003, PMID: 21889323 PMC3405730

[ref37] Lindroos B. Suuronen R. Miettinen S. (2011). The potential of adipose stem cells in regenerative medicine. Stem Cell Rev. Rep. 7, 269–291. doi: 10.1007/s12015-010-9193-7 20853072

[ref38] Liu Y. Hu Q. Dong W. Liu S. Zhang H. Gu Y. (2022). Alginate/gelatin-based hydrogel with soy protein/peptide powder for 3D printing tissue-engineering scaffolds to promote angiogenesis. Macromol. Biosci. 22:e2100413. doi: 10.1002/mabi.202100413, PMID: 35043585

[ref39] Liu Y. Yao Z. Zhang L. Zhu H. Deng W. Qin C. (2013). Insulin induces neurite outgrowth via SIRT1 in SH-SY5Y cells. Neuroscience 238, 371–380. doi: 10.1016/j.neuroscience.2013.01.034, PMID: 23357110

[ref40] Lo Furno D. Mannino G. Giuffrida R. (2018). Functional role of mesenchymal stem cells in the treatment of chronic neurodegenerative diseases. J. Cell. Physiol. 233, 3982–3999. doi: 10.1002/jcp.26192, PMID: 28926091

[ref41] López-Carballo G. Moreno L. Masiá S. Pérez P. Barettino D. (2002). Activation of the phosphatidylinositol 3-kinase/Akt signaling pathway by retinoic acid is required for neural differentiation of SH-SY5Y human neuroblastoma cells. J. Biol. Chem. 277, 25297–25304. doi: 10.1074/jbc.M201869200, PMID: 12000752

[ref42] Lou S. Gu P. Chen F. He C. Wang M. Lu C. (2003). The effect of bone marrow stromal cells on neuronal differentiation of mesencephalic neural stem cells in Sprague–Dawley rats. Brain Res. 968, 114–121. doi: 10.1016/S0006-8993(03)02224-8 12644269

[ref43] Marion N. W. Mao J. J. (2006). Mesenchymal stem cells and tissue engineering. Methods Enzymol. 420, 339–361. doi: 10.1016/S0076-6879(06)20016-8, PMID: 17161705 PMC4035029

[ref44] Miłek M. Marcinčáková D. Legáth J. (2019). Polyphenols content, antioxidant activity, and cytotoxicity assessment of *Taraxacum officinale* extracts prepared through the micelle-mediated extraction method. Molecules 24:1025. doi: 10.3390/molecules24061025, PMID: 30875865 PMC6471326

[ref45] Minor K. Tang X. Kahrilas G. Archibald S. J. Davies J. E. Davies S. J. (2008). Decorin promotes robust axon growth on inhibitory CSPGs and myelin via a direct effect on neurons. Neurobiol. Dis. 32, 88–95. doi: 10.1016/j.nbd.2008.06.009, PMID: 18638554

[ref46] Nakamura M. Nishida H. Yoshizaki K. Akiyoshi H. Hatoya S. Sugiura K.. (2020). Canine mesenchymal stromal cell-conditioned medium promotes survival and neurite outgrowth of neural stem cells. J. Vet. Med. Sci. 82, 668–672. doi: 10.1292/jvms.19-0141, PMID: 32249241 PMC7273601

[ref47] Nakano N. Nakai Y. Seo T.-B. Yamada Y. Ohno T. Yamanaka A.. (2010). Characterization of conditioned medium of cultured bone marrow stromal cells. Neurosci. Lett. 483, 57–61. doi: 10.1016/j.neulet.2010.07.062 20678542

[ref48] Negro S. Lauria F. Stazi M. Tebaldi T. D’Este G. Pirazzini M.. (2022). Hydrogen peroxide induced by nerve injury promotes axon regeneration via connective tissue growth factor. Acta Neuropathol. Commun. 10:189. doi: 10.1186/s40478-022-01495-5, PMID: 36567321 PMC9791753

[ref49] Noone C. Kihm A. English K. O’Dea S. Mahon B. P. (2013). IFN-γ stimulated human umbilical-tissue-derived cells potently suppress NK activation and resist NK-mediated cytotoxicity in vitro. Stem Cells Dev. 22, 3003–3014. doi: 10.1089/scd.2013.0028 23795941 PMC3824722

[ref50] Numakawa T. Kajihara R. (2023). Neurotrophins and other growth factors in the pathogenesis of Alzheimer’s disease. Life 13:647. doi: 10.3390/life13030647, PMID: 36983803 PMC10051261

[ref52] Park H. Y. Kim C. E. Lee S.-M. Ahn J. M. Yoon E. H. Yoo M.. (2023). Priming mesenchymal stem/stromal cells with a combination of a low dose of IFN-γ and Bortezomib results in potent suppression of pathogenic Th17 immunity through the IDO1-AHR Axis. Stem Cells 41, 64–76. doi: 10.1093/stmcls/sxac075, PMID: 36242771

[ref53] Peltzer J. Lund K. Goriot M.-E. Grosbot M. Lataillade J.-J. Mauduit P.. (2020). Interferon-γ and hypoxia priming have limited effect on the miRNA landscape of human mesenchymal stromal cells-derived extracellular vesicles. Front Cell Develop Bio 8:581436. doi: 10.3389/fcell.2020.581436, PMID: 33384991 PMC7769832

[ref54] Perez-Asensio F. J. Perpiñá U. Planas A. M. Pozas E. (2013). Interleukin-10 regulates progenitor differentiation and modulates neurogenesis in adult brain. Development 140:e2007. doi: 10.1242/dev.103812 23843621

[ref55] Petrenko Y. Vackova I. Kekulova K. Chudickova M. Koci Z. Turnovcova K.. (2020). A comparative analysis of multipotent mesenchymal stromal cells derived from different sources, with a focus on Neuroregenerative potential. Sci. Rep. 10:4290. doi: 10.1038/s41598-020-61167-z 32152403 PMC7062771

[ref56] Phinney D. G. Sensebé L. (2013). Mesenchymal stromal cells: misconceptions and evolving concepts. Cytotherapy 15, 140–145. doi: 10.1016/j.jcyt.2012.11.005, PMID: 23321325

[ref57] Pinto-Costa R. Sousa S. C. Leite S. C. Nogueira-Rodrigues J. Ferreira da Silva T. Machado D.. (2020). Profilin 1 delivery tunes cytoskeletal dynamics toward CNS axon regeneration. J. Clin. Invest. 130, 2024–2040. doi: 10.1172/JCI125771, PMID: 31945017 PMC7108904

[ref59] Qiu Z. Yang J. Deng G. Li D. Zhang S. (2021). Angiopoietin-like 4 promotes angiogenesis and neurogenesis in a mouse model of acute ischemic stroke. Brain Res. Bull. 168, 156–164. doi: 10.1016/j.brainresbull.2020.12.023, PMID: 33417949

[ref60] Rajan T. S. Diomede F. Bramanti P. Trubiani O. Mazzon E. (2017). Conditioned medium from human gingival mesenchymal stem cells protects motor-neuron-like NSC-34 cells against scratch-injury-induced cell death. Int. J. Immunopathol. Pharmacol. 30, 383–394. doi: 10.1177/0394632017740976, PMID: 29140156 PMC5806806

[ref61] Robinson K. F. Narasipura S. D. Wallace J. Ritz E. M. Al-Harthi L. (2020). Negative regulation of IL-8 in human astrocytes depends on β-catenin while positive regulation is mediated by TCFs/LEF/ATF2 interaction. Cytokine 136:155252. doi: 10.1016/j.cyto.2020.155252, PMID: 32818703 PMC7554258

[ref62] Romaus-Sanjurjo D. Saikia J. M. Kim H. J. Tsai K. M. Le G. Q. Zheng B. (2022). Overexpressing eukaryotic elongation factor 1 alpha (eEF1A) proteins to promote corticospinal axon repair after injury. Cell Death Discov. 8, 390–313. doi: 10.1038/s41420-022-01186-z, PMID: 36123349 PMC9485247

[ref63] Ryu H.-H. Kang B.-J. Park S.-S. Kim Y. Sung G.-J. Woo H.-M.. (2012). Comparison of mesenchymal stem cells derived from fat, bone marrow, Wharton’s jelly, and umbilical cord blood for treating spinal cord injuries in dogs. J. Vet. Med. Sci. 74, 1617–1630. doi: 10.1292/jvms.12-0065, PMID: 22878503

[ref64] Scheller J. Chalaris A. Schmidt-Arras D. Rose-John S. (2011). The pro- and anti-inflammatory properties of the cytokine interleukin-6. Biochimica et Biophysica Acta (BBA) - Molecular Cell Research, Including the Special Section: 11th European Symposium on Calcium 1813, 878–888. doi: 10.1016/j.bbamcr.2011.01.034 21296109

[ref65] Sharma T. P. Liu Y. Wordinger R. J. Pang I.-H. Clark A. F. (2015). Neuritin 1 promotes retinal ganglion cell survival and axonal regeneration following optic nerve crush. Cell Death Dis. 6:e1661. doi: 10.1038/cddis.2015.22, PMID: 25719245 PMC4669798

[ref66] Sheng H. Wang Y. Jin Y. Zhang Q. Zhang Y. Wang L.. (2008). A critical role of IFNγ in priming MSC-mediated suppression of T cell proliferation through up-regulation of B7-H1. Cell Res. 18, 846–857. doi: 10.1038/cr.2008.80, PMID: 18607390

[ref67] Shin S. Lee J. Kwon Y. Park K.-S. Jeong J.-H. Choi S.-J.. (2021). Comparative proteomic analysis of the mesenchymal stem cells Secretome from adipose, bone marrow, placenta and Wharton’s jelly. Int. J. Mol. Sci. 22:845. doi: 10.3390/ijms22020845, PMID: 33467726 PMC7829982

[ref68] Skaper S. D. Facci L. Zusso M. Giusti P. (2018). An inflammation-centric view of neurological disease: beyond the neuron. Front. Cell. Neurosci. 12:72. doi: 10.3389/fncel.2018.00072, PMID: 29618972 PMC5871676

[ref69] Teixeira F. G. Panchalingam K. M. Assunção-Silva R. Serra S. C. Mendes-Pinheiro B. Patrício P.. (2016). Modulation of the mesenchymal stem cell Secretome using computer-controlled bioreactors: impact on neuronal cell proliferation. Survival and Differentiation. Sci Rep 6:27791. doi: 10.1038/srep27791, PMID: 27301770 PMC4908397

[ref71] Udalamaththa V. L. Kaluarachchi A. Wijeratne S. Udagama P. V. (2020). Therapeutic uses of post-partum tissue-derived mesenchymal stromal cell secretome. Indian J. Med. Res. 152, 541–552. doi: 10.4103/ijmr.IJMR_1450_18, PMID: 34145093 PMC8224162

[ref72] Wang Y. Tu W. Lou Y. Xie A. Lai X. Guo F.. (2009). Mesenchymal stem cells regulate the proliferation and differentiation of neural stem cells through notch signaling. Cell Biol. Int. 33, 1173–1179. doi: 10.1016/j.cellbi.2009.08.004 19706332

[ref73] Wang S. Umrath F. Cen W. Salgado A. J. Reinert S. Alexander D. (2022). Pre-conditioning with IFN-γ and hypoxia enhances the Angiogenic potential of iPSC-derived MSC Secretome. Cells 11:988. doi: 10.3390/cells11060988, PMID: 35326438 PMC8946902

[ref74] Wu C.-L. Chou Y.-H. Chang Y.-J. Teng N.-Y. Hsu H.-L. Chen L. (2012). Interplay between cell migration and neurite outgrowth determines SH2B1β-enhanced neurite regeneration of differentiated PC12 cells. PLoS One 7:e34999. doi: 10.1371/journal.pone.0034999, PMID: 22539954 PMC3335126

[ref75] Wu M. Downie L. E. Grover L. M. Moakes R. J. A. Rauz S. Logan A.. (2020). The neuroregenerative effects of topical decorin on the injured mouse cornea. J. Neuroinflammation 17:142. doi: 10.1186/s12974-020-01812-6, PMID: 32366307 PMC7199348

[ref76] Wylezinski L. S. Hawiger J. (2016). Interleukin 2 activates brain microvascular endothelial cells resulting in destabilization of Adherens junctions. J. Biol. Chem. 291, 22913–22923. doi: 10.1074/jbc.M116.729038, PMID: 27601468 PMC5087713

[ref77] Yari H. Mikhailova M. V. Mardasi M. Jafarzadehgharehziaaddin M. Shahrokh S. Thangavelu L.. (2022). Emerging role of mesenchymal stromal cells (MSCs)-derived exosome in neurodegeneration-associated conditions: a groundbreaking cell-free approach. Stem Cell Res Ther 13:423. doi: 10.1186/s13287-022-03122-5, PMID: 35986375 PMC9389725

[ref78] Yun Cheng H. (2014). The impact of mesenchymal stem cell source on proliferation, differentiation, immunomodulation and therapeutic efficacy. J Stem Cell Res Ther 4:1000237. doi: 10.4172/2157-7633.1000237

[ref79] Zaim M. Karaman S. Cetin G. Isik S. (2012). Donor age and long-term culture affect differentiation and proliferation of human bone marrow mesenchymal stem cells. Ann. Hematol. 91, 1175–1186. doi: 10.1007/s00277-012-1438-x 22395436

[ref80] Zhang P. Luo X. Guo Z. Xiong A. Dong H. Zhang Q.. (2017). Neuritin inhibits notch signaling through interacted with Neuralized to promote the neurite growth. Front. Mol. Neurosci. 10:179. doi: 10.3389/fnmol.2017.00179, PMID: 28642682 PMC5462965

[ref81] Zhang K. Wang H. Xu M. Frank J. A. Luo J. (2018). Role of MCP-1 and CCR2 in ethanol-induced neuroinflammation and neurodegeneration in the developing brain. J. Neuroinflammation 15:197. doi: 10.1186/s12974-018-1241-2, PMID: 29976212 PMC6034273

[ref82] Zhang B. Yang L. Wang H. Liu M. Zhang X. Liu M.. (2019). RTCA monitors the inhibitory effect of SWCNTs on the proliferation of human liver cancer HepG2 cells. IOP Conf. Ser: Mater. Sci. Eng. 563:052070. doi: 10.1088/1757-899X/563/5/052070

[ref83] Zhou Z. Chen Y. Zhang H. Min S. Yu B. He B.. (2013). Comparison of mesenchymal stromal cells from human bone marrow and adipose tissue for the treatment of spinal cord injury. Cytotherapy 15, 434–448. doi: 10.1016/j.jcyt.2012.11.015 23376106

